# Crystal structures of an azo­benzene-bridged aza-18-crown-6 cryptand and a sodium complex thereof

**DOI:** 10.1107/S2056989026001234

**Published:** 2026-02-10

**Authors:** Christoph Schwab, Elisabeth Kreidt

**Affiliations:** a Otto-Hahn-Strasse 6A, 44227 Dortmund, Germany; Katholieke Universiteit Leuven, Belgium

**Keywords:** crystal structure, Hirshfeld surface analysis, sodium, BArF, Shinkai, crown ether, photoswitch

## Abstract

We report the single-crystal X-ray diffraction structures of the azo­benzene-bridged aza-18-crown-6 cryptand 7,16-[3,3′-(diazene-1,2-di­yl)bis­(1,3-phenyl­enecarbon­yl)]-1,4,10,13-tetra­oxa-7,16-di­aza­cyclo­octa­decane, C_26_H_32_N_4_O_6_, **1**, and its sodium complex poly[[{μ-7,16-[3,3′-(diazene-1,2-di­yl)bis­(1,3-phenyl­enecarbon­yl)]-1,4,10,13-tetra­oxa-7,16-di­aza­cyclo­octa­deca­ne}bis­{tetra­kis­[3,5-bis­(tri­fluoro­meth­yl)phen­yl]borato}disodium] benzene disolvate], {[Na_2_(C_32_H_12_F_24_B)_2_(C_26_H_32_N_4_O_6_)]·2C_6_H_6_}_*n*_. The free ligand **1** crystallizes in space group *P*2_1_/*n* and adopts an elongated strain-distorted crown conformation with a slightly twisted azo­benzene unit. In contrast, **2** [obtained with sodium tetra­kis-3,5-bis­(tri­fluoro­meth­yl)phenyl borate (NaBArF)] crystallizes in space group *P*1 and features multiple Na^+^ centers that assemble into a three-dimensional network in the solid state. Hirshfeld surface analysis was used to provide a complementary, qu­anti­tative description of the dominant inter­molecular contacts governing the crystal packing.

## Abstract 2

The single-crystal X-ray diffraction structures of an azo­benzene-bridged aza-18-crown-6 cryptand and its sodium complex are reported.

## 1. Chemical context

The azo­benzene-functionalized crown ethers reported by Shinkai and co-workers in the early 1980s (Shinkai *et al.*, 1980 ▸, 1982 ▸; Shinkai & Manabe, 1984 ▸) are considered prototypical examples of photoresponsive tools for mol­ecular recognition and transport (Hua & Flood, 2010 ▸; Hein *et al.*, 2025 ▸). They have even been referred to as the first generation of mol­ecular machines (Kinbara & Aida, 2005 ▸). As described by Shinkai and co-workers, in **1** the reversible *trans/cis* photoisomerization of the azo­benzene moiety can be used to modulate the conformation and binding properties of the 18-crown-6 cavity, allowing control of its binding affinity and selectivity towards different alkali metal cations (Shinkai *et al.*, 1980 ▸).
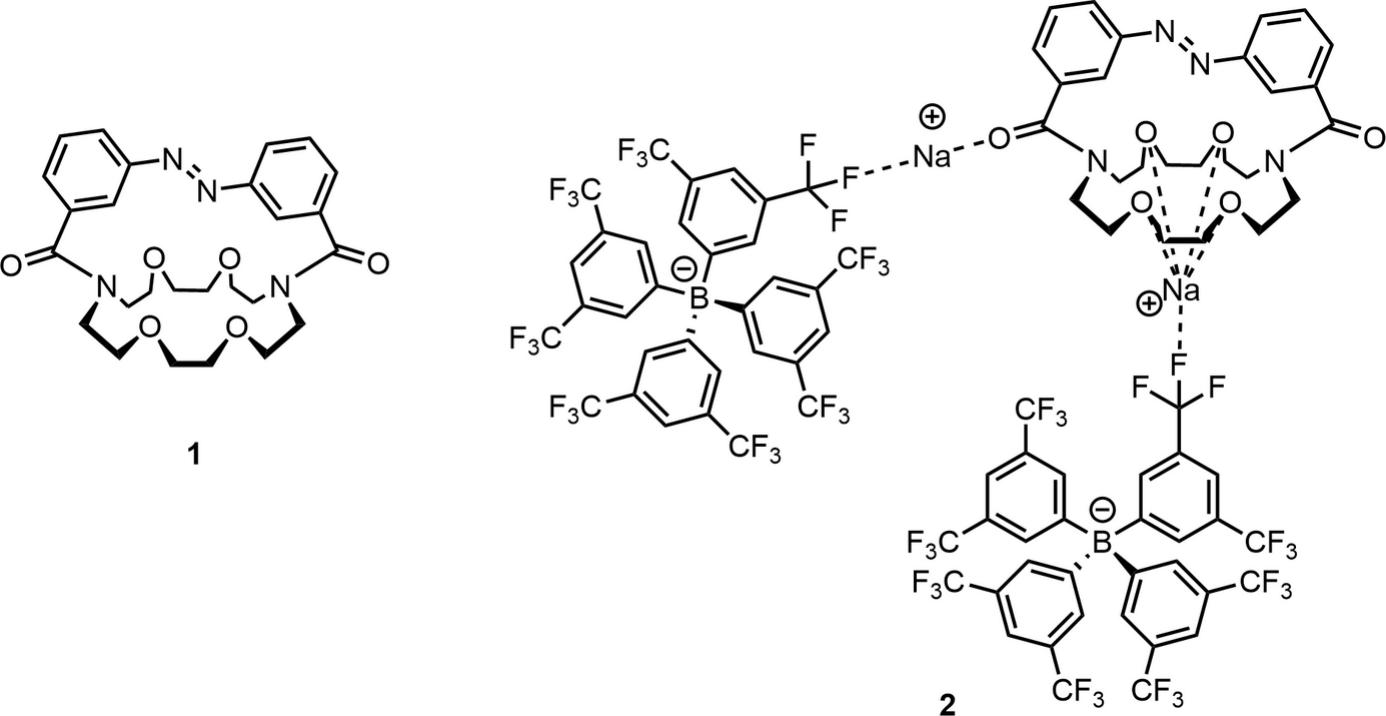


Analogous ligands with azo­pyridines and thia­crown ethers had also been described. For these also solid-state structural data are available (Shinkai *et al.*, 1984 ▸). Single-crystal X-ray diffraction studies are valuable for understanding the geometric preferences of the macrocycle, the orientation of the azo­benzene unit relative to the crown ether and, in case of the complexes, the coordination geometry of the cation. These structural insights are critical for understanding the cooperative effects between the azo­benzene moiety and the crown ether ring, and their ability to form complexes with metal cations. However, for the parent compound **1**, no solid-state structural characterization had been reported until now. In the present study, we focused on the crystallization of the free ligand **1** and its sodium complex **2** to gain a more detailed structural understanding of the cation coordination environment in the solid state.

## 2. Structural commentary

**Figure 1:**
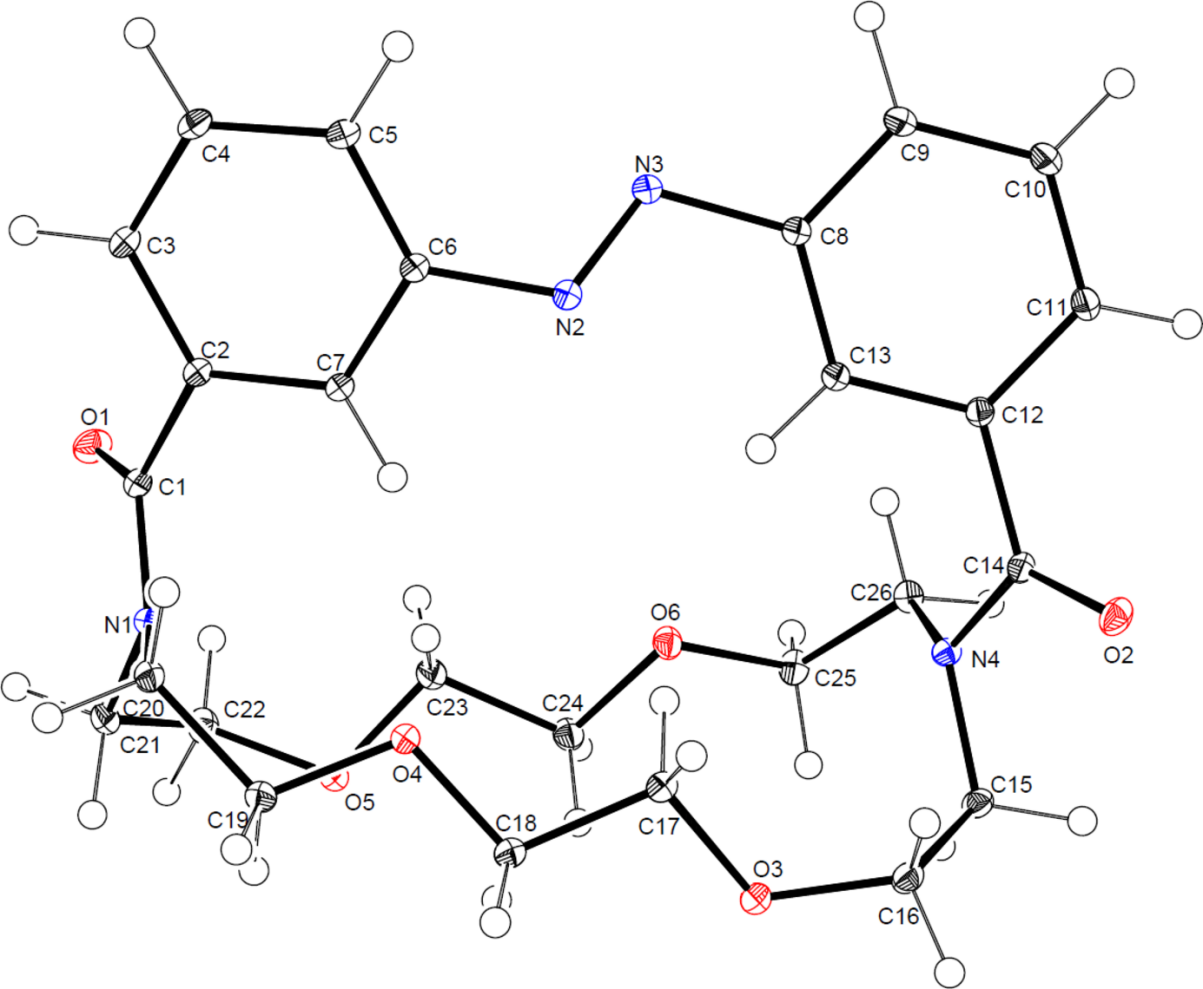
Mol­ecular structure of **1** showing the atom labelling and 50% probability ellipsoids.

Compound **1** crystallized overnight from a boiling hot toluene solution that was slowly cooled to room temperature, yielding yellow needle-shaped crystals in the centrosymmetric space group *P*2_1_*/n.* The mol­ecular structure is illustrated in Fig. 1 ▸.

Compound **1** consists of a crown ether framework (1,4,10,13-tetra­oxa-7,16-di­aza­cyclo­octa­deca­ne), whose N1 and N4 nitro­gen atoms connect via an amide bond to an azo­benzene unit, thereby forming a macrobicyclic cryptand. The amide bond lengths are 1.3629 (4) Å for the C1—N1 bond and 1.3650 (4) Å for the C14—N4 bond, which is in the normal range of amide bonds (Allen *et al.*, 1987 ▸). The *sp*^2^-hybridized nitro­gen atoms exhibit a slight pyramidalization and therefore deviate from ideal trigonal planar geometry. For N3, the pyramidalization *χ_n_*, as defined by Winkler & Dunitz (1971 ▸), is *χ_n_* = 7.12°, and for N4 it is *χ*_*n*_ = 9.33°, indicating a slight deviation from ideal planarity. The corresponding bond-angle sums are 355.81° (N3) and 352.22° (N4), values that are consistent with those expected for *sp*^2^-hybridized nitro­gen atoms (Glover & Rosser, 2018 ▸). These slight deviations are likely a consequence of intra­molecular strain.

In compound **1**, the aza­crown ether ring is significantly elongated because of the strain imposed by the azo­benzene unit. The mean distance between the opposing central carbon atoms (C24⋯C17 and C23⋯C18) is 3.6680 (6) Å, whereas the separation between the bridging nitro­gen atoms (N3 and N4) amounts to 7.5848 (5) Å. These values differ from those typically observed in unstrained aza-18-crown-6 derivates, where the distances between the opposing inward-facing donor atoms generally lie in the range of 5.4-6.0 Å (Simonov *et al.*, 2003 ▸; Chekhlov & Martynov, 1999 ▸).

The C*sp^3^*—C*sp*^3^ bond lengths within the aza­crown ether unit range from 1.5100 (5) to 1.5248 (5) Å, which are slightly shorter than the typical C—C single bond length of 1.52 Å (Allen *et al.*, 1987 ▸). The bond-angle sums around the tetra­hedral C*sp*^3^ atoms are in the range 435.81-442.90°, consistent with the values expected for *sp*^3^-hybridized carbon atoms (Allen *et al.*,1987 ▸). A gradual increase in the bond-angle sum is observed towards the bridging nitro­gen atoms, indicating a slight widening of the tetra­hedral environment in this part of the macrocycle, which may be attributed to intra­molecular strain within the aza­crown ether framework.

The Cs*p^3^*—O*sp^3^* bond lengths are in the range of 1.4194 (4)–1.4434 (5) Å, while the C—O—C bond angles are in the range 110.71 (3)–115.84 (3)°, both of which fall within the expected ranges for ether bonds (Allen *et al.*, 1987 ▸).

The azo­benzene unit is non-planar, with the two phenyl rings twisted relative to each other. The torsion angle (C—N=N—C) is −171.60 (3)°, reflecting a moderate torsion about the N=N bond. This deviation from coplanarity is best explained by intra­molecular strain within the macrocyclic framework and by crystal-packing effects. Similar distortions are also observed in the crystal structures of non-bridged azo­benzene derivatives (Strüben *et al.*, 2016 ▸).

The N2=N3 bond length is 1.2514 (4) Å, which falls within the expected range for an azo double bond of 1.23–1.26 Å (Allen *et al.*, 1987 ▸). The C—N=N bond angles of 111.81 (3) and 115.60 (3)° are close to the typical values observed in azo­benzene derivatives (Allen *et al.*, 1987 ▸; Strüben *et al.*, 2016 ▸).

Having described the structural features of the free ligand, attention may now be directed to the corresponding sodium complex (**2**), which was obtained by heating compound **1** in the presence of sodium tetra­kis-3,5-bis­(tri­fluoro­meth­yl)phenyl borate (NaBArF). Crystals suitable for X-ray diffraction were grown over three days in an NMR tube containing benzene, with approximately one-quarter of the tube immersed in an oil bath at 353 K, yielding block-like crystals in the triclinic space group *P*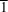.

**Figure 2:**
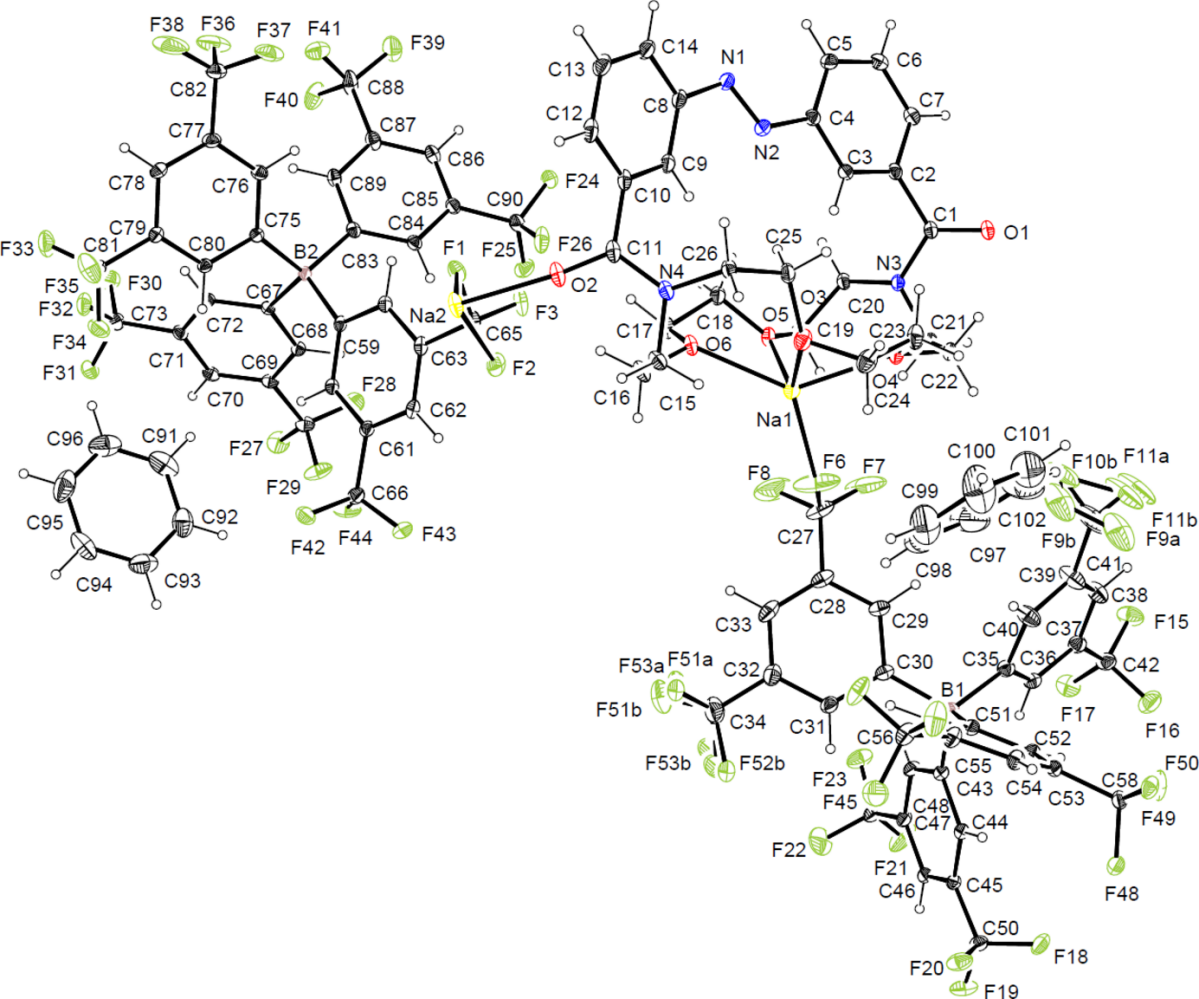
Mol­ecular structure of the sodium complex **2** showing the atom labelling and 50% probability ellipsoids.

As shown in Fig. 2 ▸, the crystal structure contains two sodium cations. One (Na2) is coordinated by four oxygen atoms from the bottom-side of the crown ether, while the second one is coordinated by the carbonyl oxygen atoms of the bridging amide functionality. Notably, the weakly coordinating BArF anions are also inter­acting with the sodium centers.

Na1 is found in a distorted octa­hedral coordination geometry with a coordination number of six, being coordinated by four oxygen atoms (O3–O6) of the crown ether ring and two fluorine atoms (F6 and F45) from two different BArF anions. The Na—O bond lengths range from 2.313 (2) to 2.366 (2) Å, while the Na—F distances are 2.362 (2) Å for Na1—F6 and 2.896 (3) Å for Na1—F45. The values for the Na—O bond lengths are slightly shorter than those reported for unstrained aza­crown ether derivatives, which typically fall in the range of 2.4–2.6 Å (Özbey *et al.*, 1998 ▸; Hu *et al.*, 2004 ▸; Rodríguez-Rodríguez *et al.*, 2014 ▸). The Na—F distances are in line with those reported in literature, which typically fall in the range of 2.3–2.9 Å (Evans *et al.*, 2023 ▸; D’Amato *et al.*, 2021 ▸; Pascu *et al.*, 2005 ▸).

Na2 has a coordination number of eight, being coordinated axially by the carbonyl oxygen atoms O1 and O2 from two different ligand mol­ecules, and by six fluorine atoms (F1, F2, F27, F28, F41, F48) from four different BArF anions. The Na—O bond lengths are 2.275 (2) Å and 2.194 (2) Å, while the Na—F contacts range from 2.482 (2) Å to 2.979 (2) Å. The values of the Na—O bond lengths are slightly shorter than those reported in the literature (Zhang *et al.*, 2011 ▸; Mahmoud *et al.*, 2019 ▸; Li *et al.*, 2015 ▸), whereas the Na—F distances are in good agreement with the literature (Evans *et al.*, 2023 ▸; D’Amato *et al.*, 2021 ▸; Pascu *et al.*, 2005 ▸).

Apart from variations in some bond angles within the macrocycle, no significant changes in bond lengths or angles are observed in comparison to the free ligand. The only notable difference concerns the torsion angle within the azo­benzene unit (C—N=N—C), with changes from −171.60 (3)° in the free ligand to −177.7 (2)° in the sodium complex. This shift towards a more planar conformation is likely a consequence of crystal-packing effects or the slight structural reorganization of the crown-ether unit induced by the sodium coordination.

## 3. Supra­molecular features

**Figure 3:**
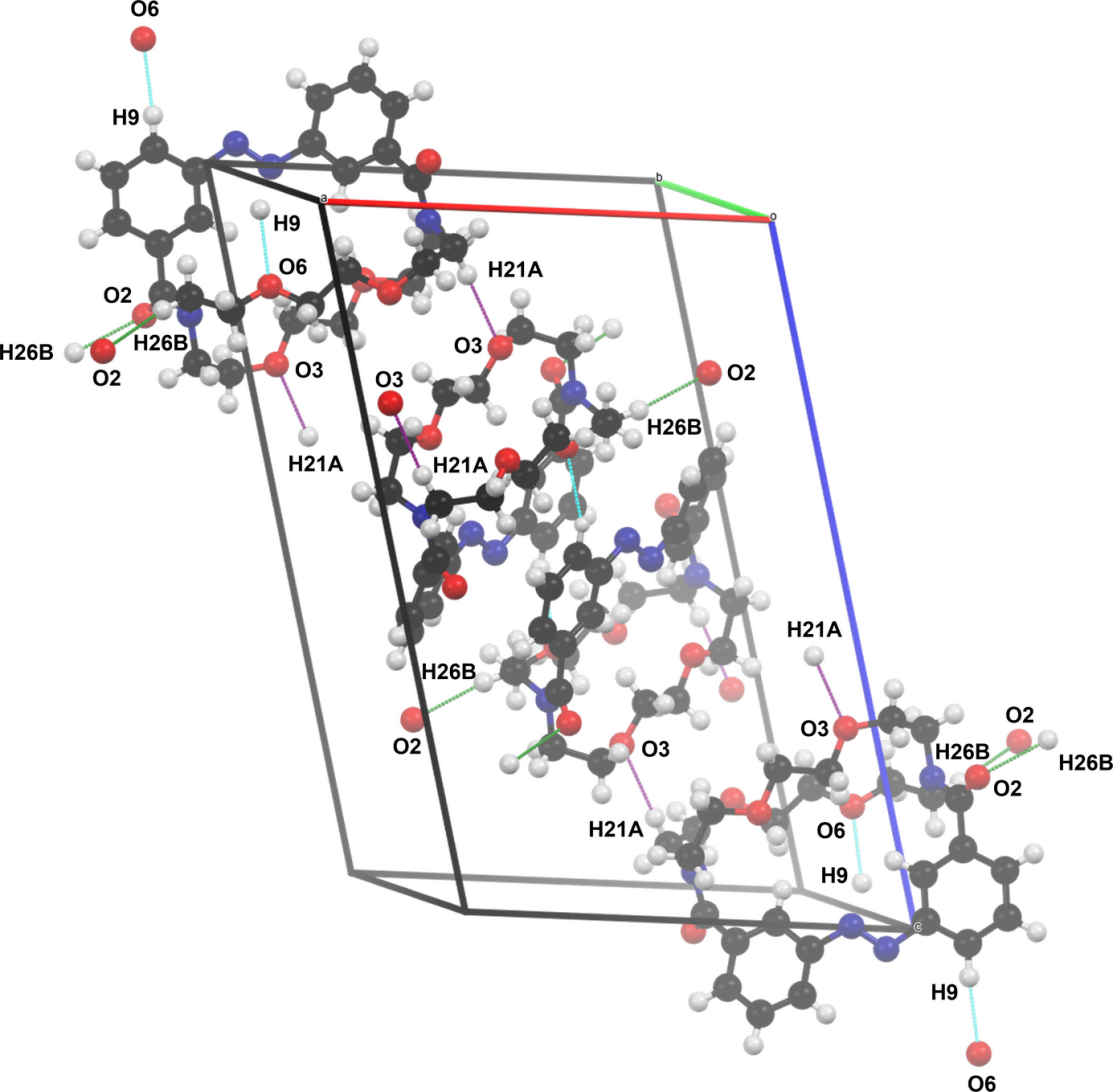
The crystal packing of compound **1**.

**Figure 4:**
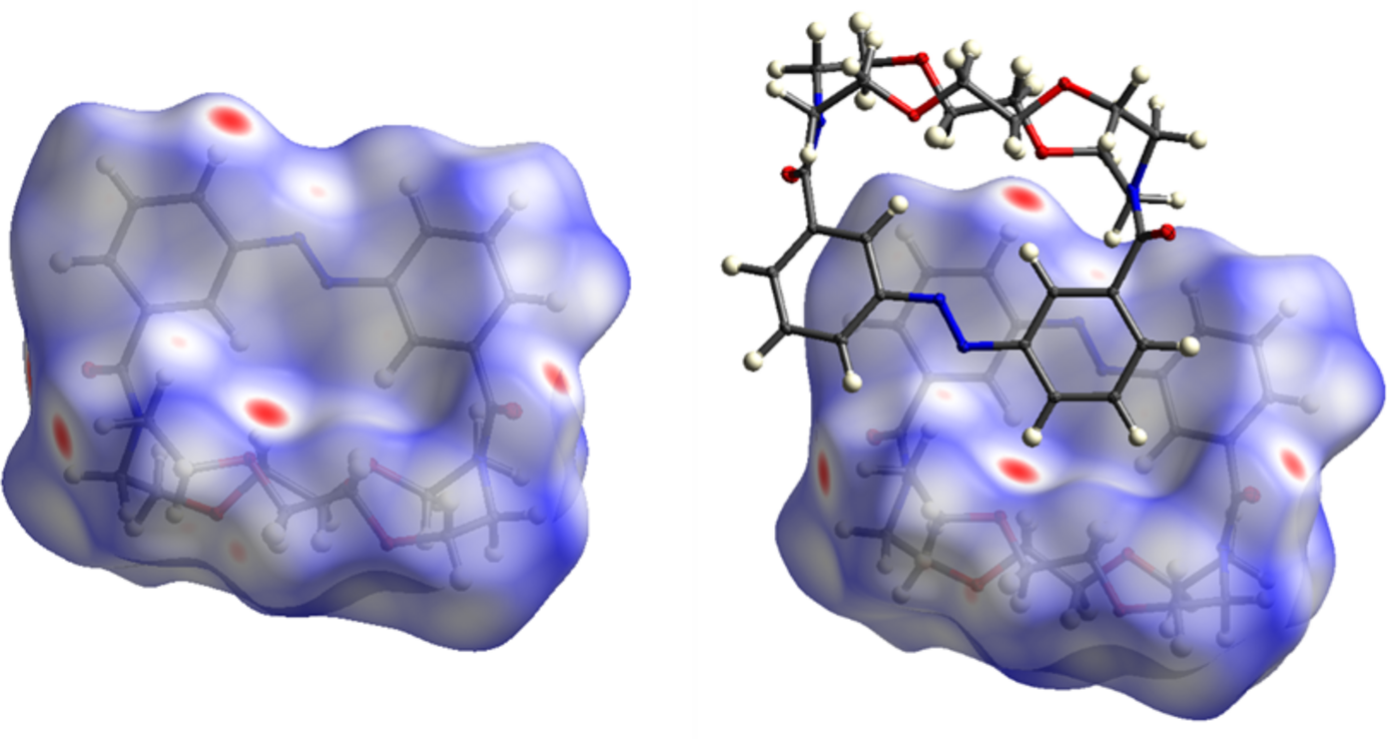
Hirshfeld surface of compound **1** (left) and Hirshfeld surface of compound **1** with a neighbouring mol­ecule, illustrating the inter­molecular contacts (right); generated by *CrystalExplorer21*.

**Figure 5:**
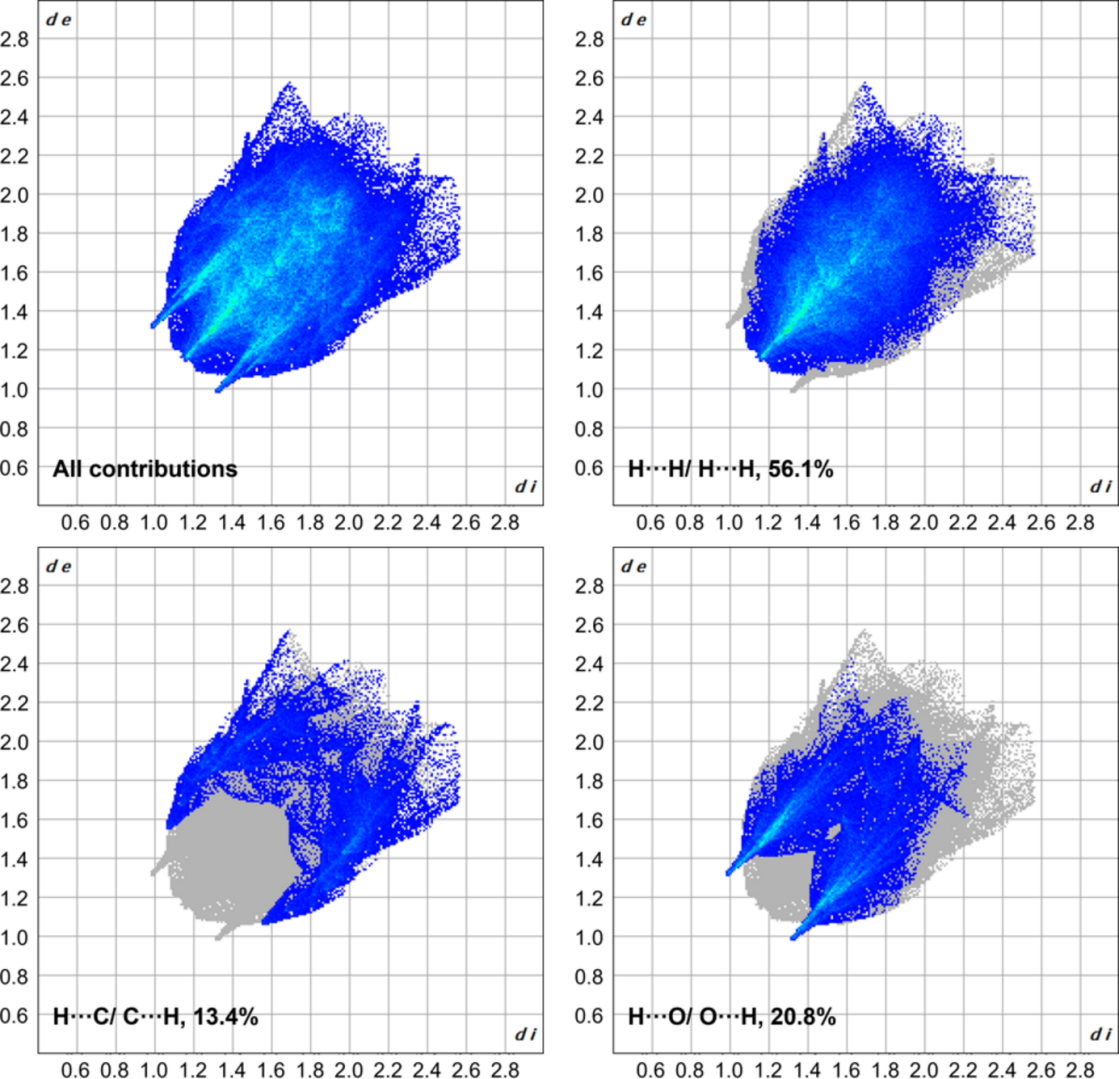
Two-dimensional fingerprint plots of compound **1** showing (*a*) all contributions in the crystal and those delineated into (*b*) H⋯H, (*c*) H⋯C/C⋯H and (*d*) H⋯O/O⋯H inter­actions.

The extended structure of compound **1** is shown in Fig. 3 ▸. The crystal packing is driven by short H⋯O contacts (Table 1 ▸), which link the mol­ecules into a chain propagating along the [100] direction. To further analyze the supra­molecular packing inter­actions, a Hirshfeld surface analysis was performed (Spackman & Jayatilaka, 2009 ▸). The Hirshfeld surface was mapped over *d*_norm_ in the range from −0.22 to 1.44 arbitrary units, generated by *CrystalExplorer21* (Spackman *et al.*, 2021 ▸) and is shown in Fig. 4 ▸. The fingerprint plots are illustrated in Fig. 5 ▸ and were also generated by *CrystalExplorer21*. Particularly notable are the short H⋯O contacts, which are shown in red on the Hirshfeld surface. They have a significant influence on the crystal packing and account for 20.8% of the overall inter­molecular inter­actions in the crystal. The H⋯H contacts (56.1%) represent the biggest fraction but contribute less significantly to the overall crystal packing.

**Figure 6:**
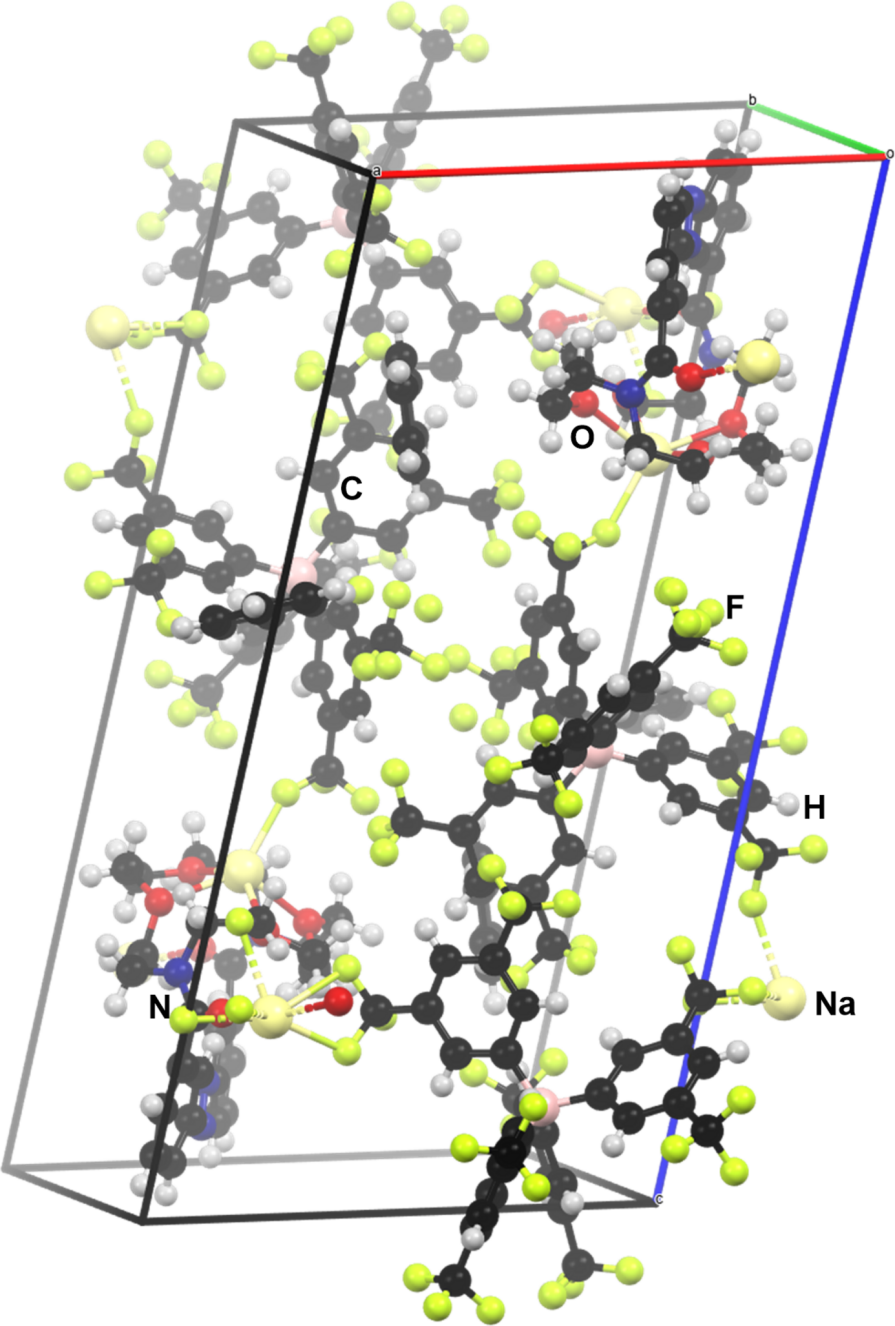
The crystal packing of the sodium complex **2**.

**Figure 7:**
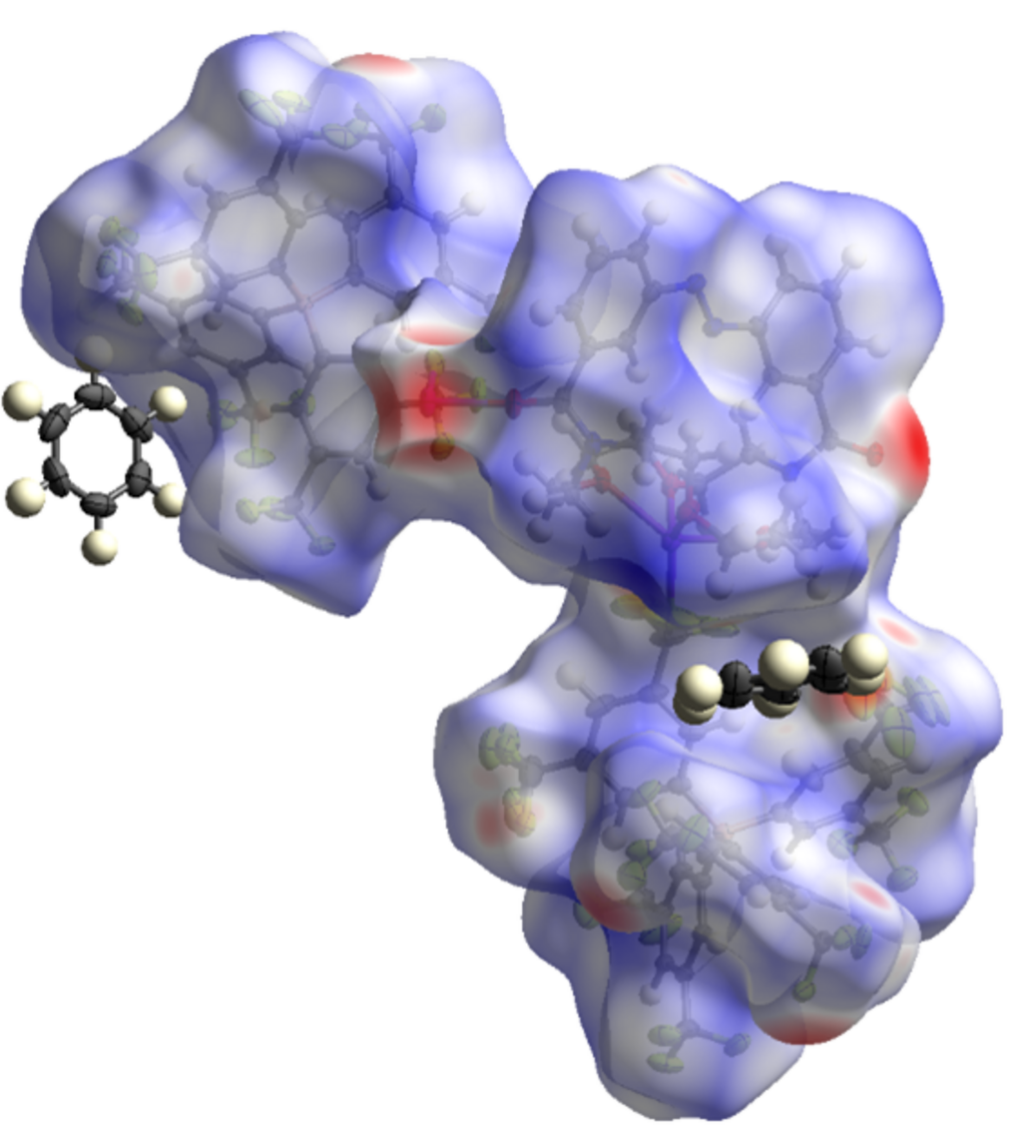
Hirshfeld surface of the sodium complex **2** generated by *CrystalExplorer21*.

**Figure 8:**
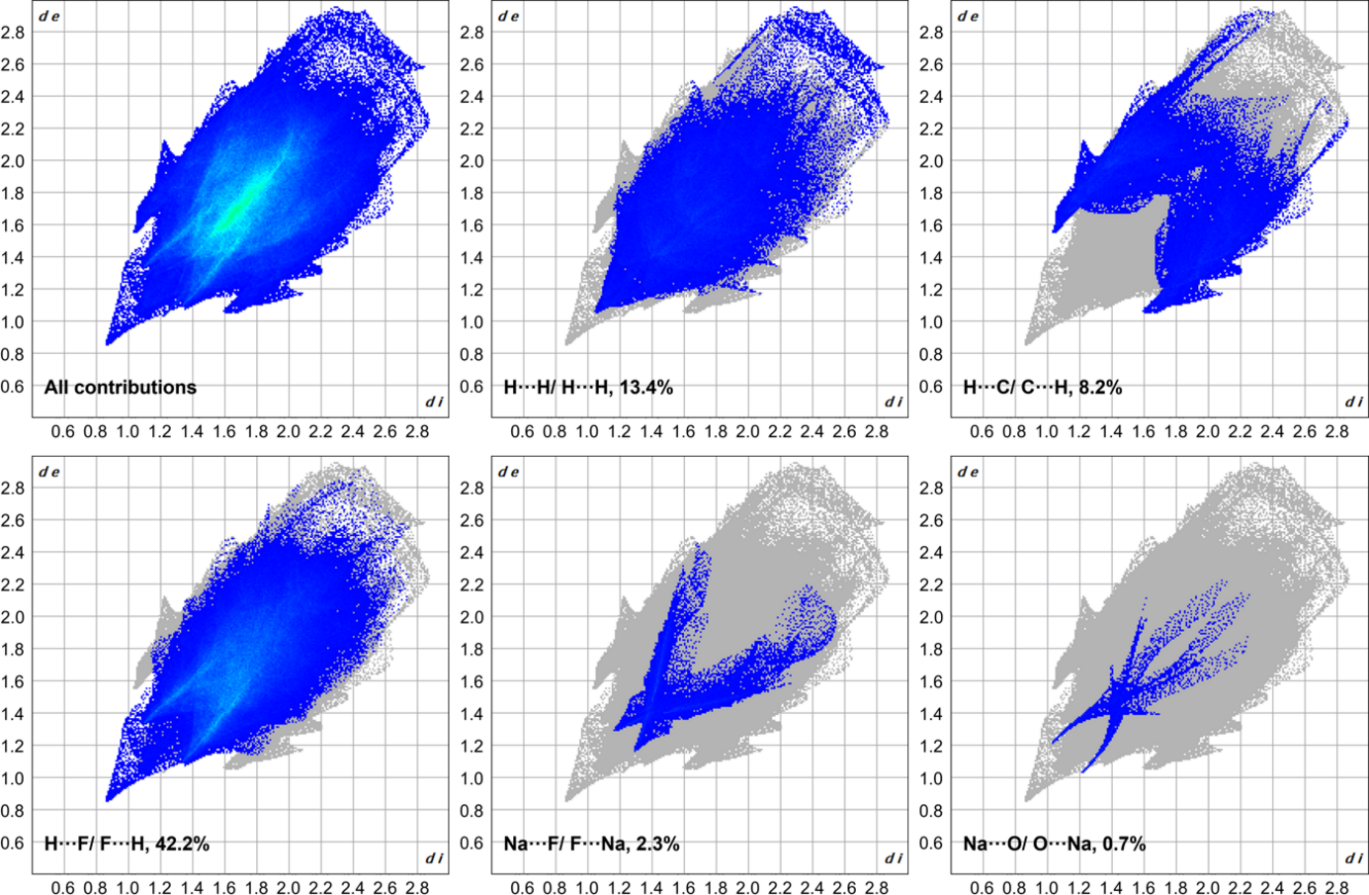
Two-dimensional fingerprint plots of the sodium complex **2** showing (*a*) all contributions in the crystal and those delineated into (*b*) H⋯H, (*c*) H⋯C/C⋯H, (*d*) H⋯F/F⋯H, (*e*) Na⋯F/F⋯Na and (*f*) Na⋯O/O⋯Na inter­actions.

For compound **2**, the extended structure is shown in Fig. 6 ▸. The packing is consolidated by Na⋯O inter­actions, involving the crown-ether and carbonyl oxygen atoms, and by Na⋯F contacts with the BArF anions. Together they generate a three-dimensional coordination-polymer framework. A Hirshfeld surface analysis (mapped over *d*_norm_ in the range from −0.73 to 2.03 arbitrary units) of the sodium complex **2** (Fig. 7 ▸) reveals that the particularly short Na⋯O and Na⋯F contacts (shown in red on the surface) exert a strong influence on the overall crystal packing, although the fingerprint plots (Fig. 8 ▸) show that these contacts represent only a small fraction of the inter­actions in the crystal (Na⋯O: 0.7%; Na⋯F: 1.3%). The largest fraction of inter­actions is represented by H⋯F contacts (42.2%), but these contribute only marginally to the overall crystal packing.

## 4. Database survey

Several crystallographically characterized structures with motifs similar to compound **1** have been reported. The following examples were identified in the Cambridge Structural Database (WebCSD, September 2025; Groom *et al.*, 2016 ▸): 1,10-(3,3′-dicarbonyl-1,1′-azo­benzene)-1,10-di­aza-4,7,14,17-tetra­thia­cyclo-octa­decane, C_26_N_4_O_2_S_4_ (CEMXAT; Ammon *et al.*, 1984 ▸), 1,10-[6,6′-dicarbonyl-2,2′-azo­pyridine-*C*(6),*C*(6′)]-1,10-di­aza-4,7,14,17-tetra­thia­cyclo-octa­decane, C_24_N_6_O_6_ (CEMXEX; Ammon *et al.*, 1984 ▸), 1,10-diazo­nia-18-crown-6 tartrate hexa­hydrate, C_16_H_20_N_2_O_16_ (HOCXIG; Chekhlov & Martynov, 1999 ▸), 1,4,10,13-tetra­oxa-7,16-di­aza­cyclo­octa­decane bis­[(1,2,5)oxa­diazolo[3,4-*d*]pyrimidine-5,7(4*H*,6*H*)-dione) clathrate dihydrate, C_28_H_34_N_10_O_12_ (BEGWAM; Simonov *et al.*, 2003 ▸) and (*E*)-[diazene-1,2-diylbis(4,1-phenyl­ene)]bis­(di­methyl­silanol) C_16_H_22_N_2_O_2_Si_2_ (OXUKAV; Strüben *et al.*, 2016 ▸).

In CEMXAT, the oxygen atoms of the crown ether ring are replaced by sulfur atoms. As expected, this substitution leads to a more flexible and less stretched macrocyclic framework compared to compound **1**. The mean distance between the centered carbon atoms is wider [5.5920 (4) Å], the distance between the bridging nitro­gen atoms remains comparable [7.8380 (7) Å]. The torsion angle of the azo­benzene unit (C—N=N—C) is larger [175.7213 (3)°] than in **1**, indicating a more planar conformation.

In CEMXEX, the azo­benzene unit is replaced by an azo­pyridine fragment. The macrocycle adopts a stretched conformation, comparable to that in **1**. The mean distance between the central opposing carbon atoms is 3.8950 (5) Å, while the separation between the bridging nitro­gen atoms amounts to 7.0906 (7) Å. The torsion angle of the azo­pyridine unit (C—N=N—C) is 173.7839 (5)°, indicating conformation similar to that in **1**.

In HOCXIG, the structure of a protonated unbridged aza­crown ether, the mean distance between the opposing oxygen atoms [5.720 (4) Å] and nitro­gen atoms [5.399 (3) Å] lie within a similar range, indicating an unstrained macrocyclic framework with a more regular, near-circular geometry compared to compound **1**.

Finally, in BEGWAM, a neutral aza­crown ether, the macrocycle also displays an unstrained and near-circular conformation. The mean distances between the opposing oxygen atoms [5.8641 (3) Å] and the opposing nitro­gen atoms [5.4364 (3) Å] lie within a comparable range.

In addition, the CSD contains entries with similar coordination environments to that observed in compound **2**, providing a useful basis for comparison: *catena*-[hexa­kis­[μ-pyridin-4-yl(2*H*-pyrrol-2-yl­idene)methano­lato]bis­(aceto­nitrile)­dimanganesedisodium aceto­nitrile solvate], C_66_H_51_Mn_2_N_15_Na_2_O_6_ (BUHSAA; Li *et al.*, 2015 ▸), (2)-[μ_2_-2,6-bis­(*n*-hex­yl)pyrrolo­[3,4-*f*]iso­indole-1,3,5,7(2*H*,6*H*)-tetrone](μ_2_-2,5,8,11,14,16,19,22,25,28-deca­oxa-1,15(1,5)-dinaphthal­ena­cyclo-octa­cosa­phane)bis­{tetra­kis­[3,5-bis­(tri­fluoro­meth­yl)phen­yl]borate-*F*,*F*′}disodiumpseudorotaxane hepta­hydrate, C_122_H_96_B_2_F_48_N_2_Na_2_O_14_ (FOFGAJ; Pascu *et al.*, 2005 ▸), (*N*,*N*′-bis­(but-3-en­yl)-1,10-di­aza-18-crown-6)sodium hexa­fluoro­phosphate, C_20_H_38_F_6_N_2_NaO_4_P (FOFGAJ; Hu *et al.*, 2004 ▸), {2,2′-[(1,4,10,13-tetra­oxa-7,16-di­aza­cyclo­octa­decane-7,16-di­yl)bis­(methyl­ene)]bis-1*H*-benzimidazole}­sodium perchlorate, C_28_H_38_N_6_O_8_NaCl (FOFRO; Rodríguez-Rodríguez *et al.*, 2014 ▸), bis­[μ-(3-{2-[1-amino-1,3-dioxobutan-2-yl­idene]hydraz­in­yl}-4-hy­droxy­benzene-1-sulfonato)]hexa­aqua­disodium, C_20_H_32_N_6_O_18_Na_2_S_2_ (FIZHUU; Mahmoud *et al.*, 2019 ▸) and *catena*-[(μ-tetra­kis­[3,5-bis­(tri­fluoro­meth­yl)phen­yl]borato)bis­(aqua)­sodium], C_32_H_16_BF_24_NaO_2_ (RENPIK01; Evans *et al.*, 2023 ▸).

In FOFGAJ, the sodium cation is located inside the cavity of the aza­crown ether and is additionally coordinated by two vinyl groups of the side arms attached to the nitro­gen atoms. The average Na—O bond length is 2.532 (2) Å, while the Na—N bond length is 2.893 (2) Å. The Na—O distance is therefore longer than that observed in **2**. In FOFROJ, where the sodium is also located within an aza­crown ether cavity, similar values are observed.

In BUHSAA, the Na—O distances of 1.264 (2) and 2.325 (1) Å are slightly longer than in **2**. The same applies for FIZHUU [2.299 (2) Å] and AQIYIJ [2.325 (3) Å].

The structures of FEKXAV and RENPIK01 contain inter­actions between a sodium cation and fluorine atoms of the CF_3_ groups of a BArF anion, similar to **2**. In FEKXAV, the Na—F distance is 2.93934 (2) Å, while in RENPIK01 the Na—F distance ranges from 2.35 (2) to 2.48 (2) Å, values that are comparable with those observed in **2**.

## 5. Synthesis and crystallization

**Figure 9:**
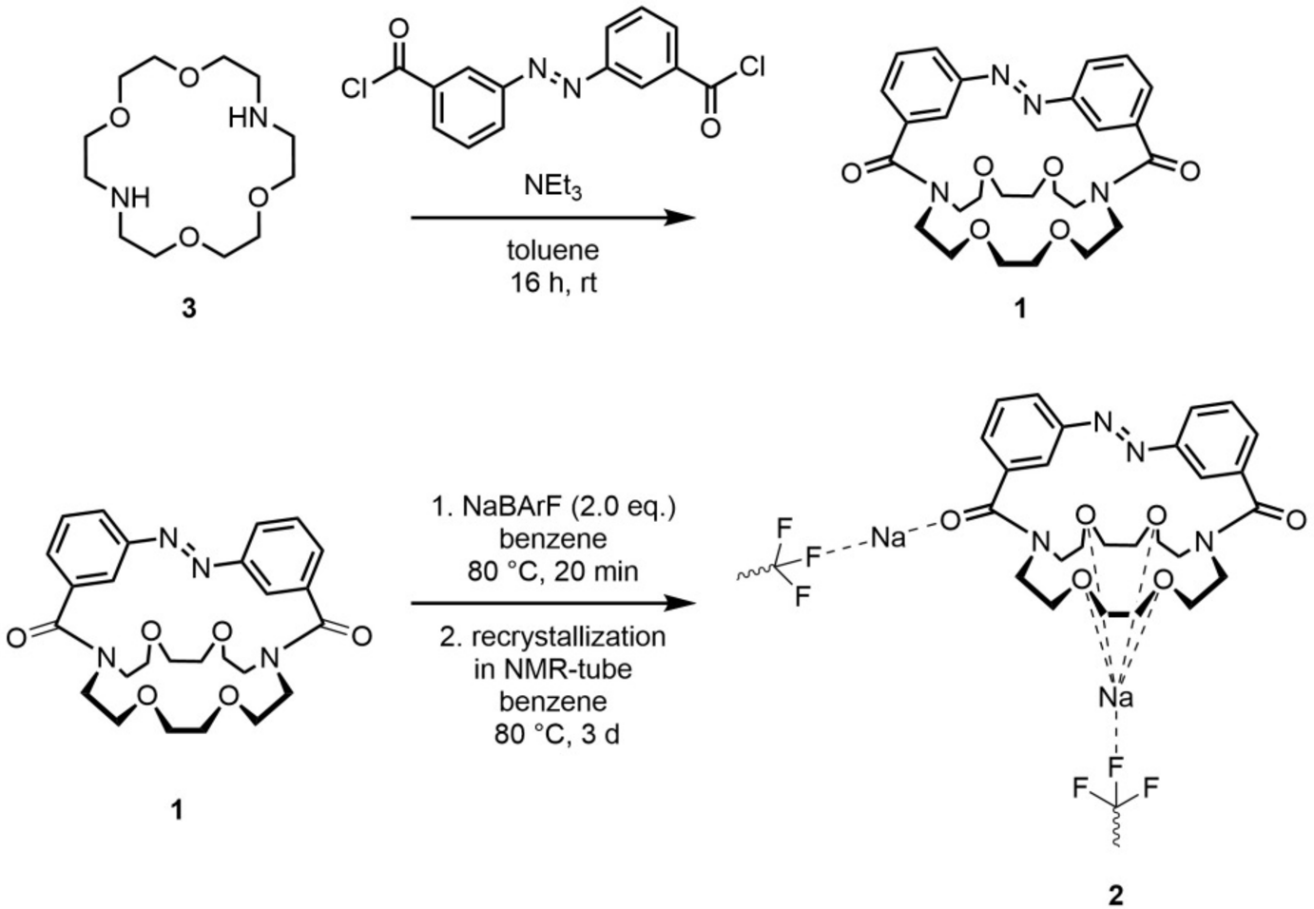
Reaction scheme for the synthesis of the ligand **1** and its sodium complex **2**.

The reaction schemes for the synthesis of compounds **1** and **2** are shown in Fig. 9 ▸.

Compound **1**, C_26_H_32_N_4_O_6_.

A solution of 3,3′-(diazene-1,2-di­yl)dibenzoyl chloride (117 mg, 0.38 mmol) in MeCN (100 mL) and a solution of 1,4,10,13-tetra­oxa-7,16-di­aza­cyclo­octa­decane (**3**, 100 mg, 0.38 mmol) in MeCN (100 mL) were added dropwise and simultaneously to a stirred solution of NEt_3_ (0.38 mL, 279 mg, 2.76 mmol) in MeCN (150 mL), preheated to 353 K. The mixture was allowed to cool to room temperature during the addition. After completion of the addition the solution was stirred for 16 h at room temperature. The solvent was removed *in vacuo* and the crude product was dissolved in CHCl_3_ and washed with water (3 × 20 mL). After removal of the volatiles, the residue was purified by silica gel column chromatography (DCM/MeOH 25:1) to yield **1** as a yellow–orange solid (135 mg, 0.27 mmol, 72%). Crystals suitable for X-ray diffraction were obtained by recrystallization from boiling hot toluene solution, giving **1** as yellow needle-shaped crystals.

^1^H-NMR (600 MHz, CDCl_3_, *δ/*ppm *J*/Hz): 8.07 (2H, *t*, *J* = 1.85, C_arom._), 7.92–7.93 (2H, *m*, C_arom_), 7.75–7.76 (2H, *m*, C_arom_), 7.60 (2H, *t*, *J* = 7.64), 3.15–4.25 (24H, *m*, CH_2_).

HRMS (ESI+) *m*/*z* calculated for C_26_H_32_N_4_O_6_: [*M*+Na]^+^ 519.2220, found 519.2216.

*Compound***2**, C_102_H_68_B_2_F_48_N_4_Na_2_O_6_.

3^4^,3^7^,3^13^,3^16^-Tetra­oxa-3^1^,3^10^,6,7-tetra­aza-3(1,10)-cyclo­octa­decana-1,5(1,3)-dibenzena­cyclo­hepta­phan-6-ene-2,4-dione (4 mg, 0.008 mmol) was dissolved in benzene (0.5 mL) in an NMR tube and NaBArF (14 mg, 0.016 mmol) was added. The mixture was heated at 353 K for 15 min, during which the solution became colorless and an orange solid precipitated. The tube was then filled to approximately five-sixth with benzene and placed to about one-quarter of its length in an oil bath kept at 353 K. Crystals of the sodium complex **2**, suitable for X-ray diffraction, were obtained after three days as yellow block-like crystals in the upper part of the tube.

## 6. Refinement

Crystal data, data collection and structure refinement details are summarized in Table 2 ▸. Hydrogen atoms were positioned geometrically and refined using a riding model with *U*_iso_(H) set to 1.2*U*_eq_ of the carrier atom (1.5*U*_eq_ for methyl groups). Two CF_3_ groups are rotationally disordered about the C—CF_3_ bonds: at C34 (sets F51/F2/F4 and F1/F3/F10) with ratio 0.651 (4):0.349 (4), and at C41 (sets F11/F14/F9 and F12/F13/F10) with ratio 0.771 (6):0.229 (6). The disorder was modeled with PART instructions; distance-similarity restraints (SADI) and displacement-parameter restraints (RIGU, SIMU, ISOR) were applied to maintain reasonable geometry and ADPs for the disordered atoms.

## Supplementary Material

Crystal structure: contains datablock(s) 1, 2. DOI: 10.1107/S2056989026001234/vm2322sup1.cif

Structure factors: contains datablock(s) 1. DOI: 10.1107/S2056989026001234/vm23221sup2.hkl

Structure factors: contains datablock(s) 2. DOI: 10.1107/S2056989026001234/vm23222sup3.hkl

CCDC references: 2528906, 2528905

Additional supporting information:  crystallographic information; 3D view; checkCIF report

## Figures and Tables

**Table 1:** Geometric parameters (Å, °) and chain directions for selected C—H⋯O contacts

	C21—H21*A*⋯O3	C26—H26*B*⋯O2	C9—H9⋯O6
*D*—H	1.012 (11)	1.005 (11)	1.008 (11)
H⋯*A*	2.498 (11)	2.395 (11)	2.405 (11)
*D*⋯*A*	3.5028 (5)	3.3917 (5)	3.3677 (4)
*D*—H⋯*A*	171.7 (8)	171.3 (9)	159.6 (9)
Direction of chain	[100]	[001]	[100]

**Table 2:** Experimental details

	**1**	**2**
Crystal data		
Chemical formula	C_26_H_32_N_4_O_6_	[Na_2_(C_32_H_12_F_24_B)_2_(C_26_H_32_N_4_O_6_)]·2C_6_H_6_
*M*_r_	496.57	2425.20
Crystal system, space group	Monoclinic, *P*2_1_/*n*	Triclinic, *P* 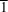
Temperature (K)	100	100
*a*, *b*, *c* (Å)	14.9099 (7), 7.7078 (3), 22.1751 (10)	13.5857 (6), 13.8054 (6), 27.8552 (11)
α, β, γ (°)	90, 108.751 (1), 90	84.152 (2), 78.185 (2), 89.238 (2)
*V* (Å^3^)	2413.16 (18)	5087.0 (4)
*Z*	4	2
Radiation type	Ag *K*α, λ = 0.56086 Å	Cu *K*α
μ (mm^−1^)	0.06	1.49
Crystal size (mm)	0.8 × 0.3 × 0.2	0.30 × 0.29 × 0.24

Data collection		
Diffractometer	Bruker APEXII CCD	Bruker APEXII CCD
Absorption correction	Multi-scan (*SADABS*; Krause *et al.*, 2015 ▸)	Multi-scan (*SADABS*; Krause *et al.*, 2015 ▸)
*T*_min_, *T*_max_	0.408, 0.435	0.342, 0.471
No. of measured, independent and observed reflections	1669726, 27661, 24282 [*I* ≥ 2u(*I*)]	168897, 20857, 18230 [*I* > 2σ(*I*)]
*R*_int_	0.065	0.044
(sin θ/λ)_max_ (Å^−1^)	1.111	0.626

Refinement		
*R*[*F*^2^ > 2σ(*F*^2^)], *wR*(*F*^2^), *S*	0.040, 0.115, 1.04	0.071, 0.205, 1.08
No. of reflections	27661	20857
No. of parameters	453	1473
No. of restraints	0	244
H-atom treatment	All H-atom parameters refined	H-atom parameters constrained
Δρ_max_, Δρ_min_ (e Å^−3^)	0.66, −0.33	1.45, −0.76

Computer programs: *APEX4* and *SAINT* (Bruker, 2016 ▸), *OLEX2.solve* and *OLEX2.refine* (Bourhis *et al.*, 2015 ▸), *SHELXT* (Sheldrick, 2015*a* ▸), *SHELXL* (Sheldrick, 2015*b* ▸) and *OLEX2* (Dolomanov *et al.*, 2009 ▸).

## supplementary crystallographic information

### 7,16-[3,3'-(Diazene-1,2-diyl)bis(1,3-phenylenecarbonyl)]-1,4,10,13-tetraoxa-7,16-diazacyclooctadecane (1) Crystal data

**Table:**

C_26_H_32_N_4_O_6_	*F*(000) = 1056.322
*M**_r_* = 496.57	*D*_x_ = 1.367 Mg m^−^^3^
Monoclinic, *P*2_1_/*n*	Ag *K*α radiation, λ = 0.56086 Å
*a* = 14.9099 (7) Å	Cell parameters from 9994 reflections
*b* = 7.7078 (3) Å	θ = 2.2–18.1°
*c* = 22.1751 (10) Å	µ = 0.06 mm^−^^1^
β = 108.751 (1)°	*T* = 100 K
*V* = 2413.16 (18) Å^3^	Needle, yellow
*Z* = 4	0.8 × 0.3 × 0.2 mm

### 7,16-[3,3'-(Diazene-1,2-diyl)bis(1,3-phenylenecarbonyl)]-1,4,10,13-tetraoxa-7,16-diazacyclooctadecane (1) Data collection

**Table:**

Bruker APEXII CCD diffractometer	24282 reflections with *I*≥ 2u(*I*)
Detector resolution: 7.9 pixels mm^-1^	*R*_int_ = 0.065
φ and ω scans	θ_max_ = 38.5°, θ_min_ = 1.5°
Absorption correction: multi-scan (SADABS; Krause *et al.*, 2015)	*h* = −33→33
*T*_min_ = 0.408, *T*_max_ = 0.435	*k* = −17→17
1669726 measured reflections	*l* = −49→49
27661 independent reflections	

### 7,16-[3,3'-(Diazene-1,2-diyl)bis(1,3-phenylenecarbonyl)]-1,4,10,13-tetraoxa-7,16-diazacyclooctadecane (1) Refinement

**Table:**

Refinement on *F*^2^	0 constraints
Least-squares matrix: full	Primary atom site location: iterative
*R*[*F*^2^ > 2σ(*F*^2^)] = 0.040	All H-atom parameters refined
*wR*(*F*^2^) = 0.115	*w* = 1/[σ^2^(*F*_o_^2^) + (0.0585*P*)^2^ + 0.3262*P*] where *P* = (*F*_o_^2^ + 2*F*_c_^2^)/3
*S* = 1.04	(Δ/σ)_max_ = 0.001
27661 reflections	Δρ_max_ = 0.66 e Å^−^^3^
453 parameters	Δρ_min_ = −0.33 e Å^−^^3^
0 restraints	

### 7,16-[3,3'-(Diazene-1,2-diyl)bis(1,3-phenylenecarbonyl)]-1,4,10,13-tetraoxa-7,16-diazacyclooctadecane (1) Fractional atomic coordinates and isotropic or equivalent isotropic displacement parameters (Å^2^)

**Table:**

	*x*	*y*	*z*	*U*_iso_*/*U*_eq_	
O3	0.55481 (2)	0.46129 (4)	0.219451 (13)	0.01385 (4)	
O6	0.514125 (19)	0.20077 (4)	0.342646 (14)	0.01290 (4)	
O4	0.771280 (19)	0.39051 (4)	0.347818 (12)	0.01249 (4)	
O5	0.71583 (2)	−0.06721 (4)	0.353696 (14)	0.01520 (4)	
O2	0.36419 (2)	0.80557 (4)	0.264270 (14)	0.01417 (4)	
O1	0.87734 (3)	−0.00660 (4)	0.539160 (15)	0.01636 (5)	
N4	0.39753 (2)	0.51839 (4)	0.282315 (13)	0.00963 (3)	
N2	0.66862 (2)	0.61248 (4)	0.492513 (14)	0.01188 (4)	
N1	0.88028 (2)	0.12433 (4)	0.448014 (13)	0.01081 (4)	
N3	0.61260 (2)	0.66152 (4)	0.520678 (14)	0.01133 (4)	
C12	0.40783 (2)	0.71471 (4)	0.371663 (15)	0.00998 (4)	
C8	0.52280 (2)	0.71185 (4)	0.476844 (15)	0.01011 (4)	
C2	0.86078 (2)	0.29794 (4)	0.536550 (15)	0.01067 (4)	
C14	0.38541 (2)	0.68225 (4)	0.301726 (15)	0.00983 (4)	
C11	0.34626 (2)	0.81097 (5)	0.394823 (17)	0.01231 (4)	
C15	0.38848 (2)	0.50407 (5)	0.214721 (15)	0.01147 (4)	
C9	0.46051 (2)	0.80144 (5)	0.500862 (16)	0.01223 (4)	
C6	0.75456 (2)	0.53760 (4)	0.531792 (15)	0.01057 (4)	
C7	0.78606 (2)	0.40326 (5)	0.501719 (15)	0.01114 (4)	
C10	0.37218 (2)	0.85171 (5)	0.459331 (18)	0.01338 (5)	
C26	0.36959 (2)	0.36854 (4)	0.313840 (16)	0.01113 (4)	
C1	0.87632 (2)	0.12759 (5)	0.508558 (15)	0.01114 (4)	
C3	0.90911 (2)	0.33823 (5)	0.600311 (16)	0.01242 (4)	
C13	0.49581 (2)	0.66452 (4)	0.412558 (15)	0.01082 (4)	
C19	0.85884 (3)	0.31131 (5)	0.351561 (17)	0.01328 (5)	
C5	0.80275 (2)	0.58062 (5)	0.595111 (16)	0.01229 (4)	
C20	0.91627 (2)	0.27134 (5)	0.420240 (16)	0.01183 (4)	
C4	0.88167 (3)	0.48247 (5)	0.628526 (16)	0.01325 (5)	
C18	0.71091 (2)	0.39965 (5)	0.283500 (16)	0.01310 (5)	
C16	0.47464 (2)	0.57375 (5)	0.199716 (16)	0.01262 (4)	
C23	0.65659 (3)	0.03910 (5)	0.377230 (18)	0.01458 (5)	
C17	0.62048 (2)	0.49376 (5)	0.281401 (16)	0.01219 (4)	
C24	0.56377 (3)	0.06357 (5)	0.324276 (18)	0.01334 (5)	
C21	0.88415 (2)	−0.04816 (5)	0.420429 (18)	0.01286 (4)	
C25	0.41595 (3)	0.19833 (4)	0.307544 (18)	0.01305 (5)	
C22	0.79114 (3)	−0.14859 (5)	0.40218 (2)	0.01460 (5)	
H26a	0.3841 (7)	0.3932 (12)	0.3586 (5)	0.018 (2)*	
H17a	0.5966 (7)	0.4510 (12)	0.3156 (5)	0.018 (2)*	
H20a	0.9832 (7)	0.2423 (13)	0.4216 (4)	0.0174 (19)*	
H15a	0.3814 (7)	0.3807 (12)	0.2031 (4)	0.0159 (19)*	
H13	0.5396 (7)	0.5974 (13)	0.3980 (5)	0.021 (2)*	
H5	0.7806 (7)	0.6765 (13)	0.6160 (5)	0.022 (2)*	
H24a	0.5751 (8)	0.0947 (14)	0.2843 (5)	0.025 (2)*	
H7	0.7512 (7)	0.3784 (13)	0.4568 (5)	0.021 (2)*	
H16a	0.4916 (7)	0.6843 (13)	0.2190 (5)	0.018 (2)*	
H15b	0.3320 (7)	0.5691 (13)	0.1877 (5)	0.019 (2)*	
H11	0.2830 (7)	0.8464 (14)	0.3678 (5)	0.025 (2)*	
H21a	0.9051 (7)	−0.0336 (13)	0.3816 (5)	0.021 (2)*	
H20b	0.9205 (7)	0.3730 (12)	0.4479 (4)	0.017 (2)*	
H19a	0.8471 (7)	0.2078 (13)	0.3249 (5)	0.021 (2)*	
H9	0.4790 (7)	0.8261 (14)	0.5479 (5)	0.023 (2)*	
H17b	0.6319 (7)	0.6175 (13)	0.2876 (5)	0.020 (2)*	
H18a	0.7431 (8)	0.4558 (14)	0.2590 (5)	0.026 (2)*	
H26b	0.2991 (7)	0.3530 (12)	0.2951 (5)	0.0164 (19)*	
H19b	0.8978 (7)	0.3895 (13)	0.3338 (5)	0.021 (2)*	
H16b	0.4584 (7)	0.5834 (13)	0.1548 (5)	0.020 (2)*	
H4	0.9183 (7)	0.5114 (14)	0.6721 (5)	0.022 (2)*	
H23a	0.6445 (8)	−0.0198 (14)	0.4144 (5)	0.026 (2)*	
H10	0.3293 (7)	0.9177 (12)	0.4755 (5)	0.019 (2)*	
H23b	0.6863 (8)	0.1538 (14)	0.3909 (5)	0.028 (3)*	
H24b	0.5271 (8)	−0.0452 (15)	0.3178 (5)	0.028 (2)*	
H21b	0.9323 (7)	−0.1188 (13)	0.4526 (5)	0.019 (2)*	
H22a	0.7717 (7)	−0.1685 (14)	0.4396 (5)	0.025 (2)*	
H25a	0.3844 (7)	0.1060 (13)	0.3239 (5)	0.020 (2)*	
H18b	0.6953 (7)	0.2842 (14)	0.2675 (5)	0.024 (2)*	
H3	0.9626 (7)	0.2633 (14)	0.6260 (5)	0.023 (2)*	
H22b	0.8009 (8)	−0.2615 (15)	0.3827 (5)	0.029 (3)*	
H25b	0.4077 (7)	0.1686 (12)	0.2626 (4)	0.017 (2)*	

### 7,16-[3,3'-(Diazene-1,2-diyl)bis(1,3-phenylenecarbonyl)]-1,4,10,13-tetraoxa-7,16-diazacyclooctadecane (1) Atomic displacement parameters (Å^2^)

**Table:**

	*U*^11^	*U*^22^	*U*^33^	*U*^12^	*U*^13^	*U*^23^
O3	0.01159 (8)	0.01825 (10)	0.01036 (8)	0.00223 (7)	0.00163 (6)	−0.00202 (7)
O6	0.01144 (8)	0.01153 (8)	0.01427 (9)	0.00090 (6)	0.00208 (7)	−0.00249 (7)
O4	0.01149 (8)	0.01642 (10)	0.00939 (7)	0.00372 (7)	0.00311 (6)	0.00088 (7)
O5	0.01271 (9)	0.01924 (11)	0.01301 (9)	0.00376 (8)	0.00327 (7)	−0.00292 (8)
O2	0.01719 (10)	0.01029 (8)	0.01243 (9)	0.00170 (7)	0.00114 (7)	0.00233 (7)
O1	0.02231 (12)	0.01391 (10)	0.01229 (9)	0.00253 (9)	0.00478 (8)	0.00345 (7)
N4	0.01076 (8)	0.00882 (8)	0.00912 (8)	−0.00035 (6)	0.00292 (6)	−0.00009 (6)
N2	0.01041 (8)	0.01532 (10)	0.00953 (8)	0.00245 (7)	0.00266 (6)	−0.00006 (7)
N1	0.01111 (8)	0.01178 (9)	0.00971 (8)	0.00118 (7)	0.00357 (6)	−0.00012 (7)
N3	0.01062 (8)	0.01365 (9)	0.00939 (8)	0.00127 (7)	0.00277 (6)	−0.00112 (7)
C12	0.00969 (8)	0.00974 (9)	0.00990 (9)	0.00075 (7)	0.00231 (7)	−0.00055 (7)
C8	0.00983 (8)	0.01099 (9)	0.00939 (9)	0.00076 (7)	0.00295 (7)	−0.00092 (7)
C2	0.01023 (9)	0.01293 (10)	0.00819 (8)	0.00101 (7)	0.00202 (7)	0.00000 (7)
C14	0.00937 (8)	0.00929 (9)	0.00971 (9)	0.00038 (7)	0.00148 (7)	0.00022 (7)
C11	0.01027 (9)	0.01296 (10)	0.01344 (10)	0.00226 (8)	0.00344 (8)	−0.00048 (8)
C15	0.01093 (9)	0.01344 (10)	0.00890 (9)	−0.00083 (8)	0.00159 (7)	−0.00095 (8)
C9	0.01238 (10)	0.01384 (11)	0.01130 (10)	0.00113 (8)	0.00496 (8)	−0.00172 (8)
C6	0.00988 (9)	0.01269 (10)	0.00867 (8)	0.00081 (7)	0.00230 (7)	−0.00039 (7)
C7	0.01035 (9)	0.01413 (11)	0.00806 (8)	0.00185 (8)	0.00173 (7)	−0.00072 (8)
C10	0.01194 (10)	0.01511 (12)	0.01411 (11)	0.00242 (9)	0.00563 (8)	−0.00170 (9)
C26	0.01048 (9)	0.00992 (9)	0.01307 (10)	−0.00087 (7)	0.00388 (8)	0.00067 (8)
C1	0.01086 (9)	0.01265 (10)	0.00912 (9)	0.00178 (8)	0.00210 (7)	0.00076 (8)
C3	0.01186 (10)	0.01500 (11)	0.00877 (9)	0.00089 (8)	0.00103 (7)	0.00038 (8)
C13	0.00998 (9)	0.01231 (10)	0.00956 (9)	0.00182 (7)	0.00230 (7)	−0.00146 (7)
C19	0.01242 (10)	0.01732 (12)	0.01097 (10)	0.00324 (9)	0.00497 (8)	0.00201 (9)
C5	0.01255 (10)	0.01395 (11)	0.00942 (9)	−0.00016 (8)	0.00219 (8)	−0.00215 (8)
C20	0.01002 (9)	0.01432 (11)	0.01126 (10)	0.00075 (8)	0.00357 (7)	0.00080 (8)
C4	0.01333 (10)	0.01575 (12)	0.00857 (9)	−0.00039 (9)	0.00058 (8)	−0.00149 (8)
C18	0.01208 (10)	0.01771 (12)	0.00958 (9)	0.00204 (9)	0.00358 (8)	0.00135 (9)
C16	0.01191 (10)	0.01590 (12)	0.00972 (9)	0.00084 (8)	0.00299 (8)	0.00245 (8)
C23	0.01311 (11)	0.01623 (12)	0.01355 (11)	0.00248 (9)	0.00311 (9)	−0.00316 (9)
C17	0.01145 (9)	0.01481 (11)	0.00978 (9)	0.00106 (8)	0.00266 (8)	0.00021 (8)
C24	0.01191 (10)	0.01279 (11)	0.01468 (11)	−0.00001 (8)	0.00336 (8)	−0.00290 (9)
C21	0.01191 (10)	0.01310 (11)	0.01366 (11)	0.00267 (8)	0.00421 (8)	−0.00119 (9)
C25	0.01228 (10)	0.00948 (9)	0.01554 (11)	−0.00091 (8)	0.00190 (9)	−0.00062 (8)
C22	0.01394 (11)	0.01178 (11)	0.01712 (13)	0.00144 (9)	0.00366 (9)	−0.00079 (9)

### 7,16-[3,3'-(Diazene-1,2-diyl)bis(1,3-phenylenecarbonyl)]-1,4,10,13-tetraoxa-7,16-diazacyclooctadecane (1) Geometric parameters (Å, º)

**Table:**

O3—C16	1.4266 (5)	C7—H7	0.983 (10)
O3—C17	1.4296 (4)	C10—H10	0.971 (10)
O6—C24	1.4234 (5)	C26—C25	1.5100 (5)
O6—C25	1.4200 (4)	C26—H26a	0.964 (10)
O4—C19	1.4193 (4)	C26—H26b	1.006 (9)
O4—C18	1.4227 (4)	C3—C4	1.3998 (5)
O5—C23	1.4213 (5)	C3—H3	1.001 (10)
O5—C22	1.4262 (5)	C13—H13	0.967 (10)
O2—C14	1.2347 (4)	C19—C20	1.5196 (5)
O1—C1	1.2344 (5)	C19—H19a	0.975 (10)
N4—C14	1.3650 (4)	C19—H19b	1.001 (10)
N4—C15	1.4653 (4)	C5—C4	1.3949 (5)
N4—C26	1.4777 (4)	C5—H5	0.984 (10)
N2—N3	1.2514 (4)	C20—H20a	1.014 (9)
N2—C6	1.4199 (4)	C20—H20b	0.985 (9)
N1—C1	1.3629 (4)	C4—H4	0.970 (10)
N1—C20	1.4709 (5)	C18—C17	1.5185 (5)
N1—C21	1.4724 (5)	C18—H18a	0.938 (11)
N3—C8	1.4323 (4)	C18—H18b	0.959 (11)
C12—C14	1.4988 (4)	C16—H16a	0.950 (10)
C12—C11	1.4000 (5)	C16—H16b	0.949 (10)
C12—C13	1.3886 (4)	C23—C24	1.5116 (5)
C8—C9	1.3932 (4)	C23—H23a	1.007 (11)
C8—C13	1.3995 (4)	C23—H23b	0.991 (11)
C2—C7	1.3941 (4)	C17—H17a	0.992 (10)
C2—C1	1.5019 (5)	C17—H17b	0.970 (10)
C2—C3	1.3999 (4)	C24—H24a	0.984 (11)
C11—C10	1.3924 (5)	C24—H24b	0.986 (11)
C11—H11	0.980 (11)	C21—C22	1.5249 (5)
C15—C16	1.5245 (5)	C21—H21a	1.012 (10)
C15—H15a	0.982 (9)	C21—H21b	0.995 (10)
C15—H15b	0.998 (10)	C25—H25a	0.985 (10)
C9—C10	1.3975 (5)	C25—H25b	0.991 (9)
C9—H9	1.008 (10)	C22—H22a	0.975 (11)
C6—C7	1.3924 (5)	C22—H22b	1.003 (11)
C6—C5	1.3961 (4)		
			
C17—O3—C16	115.85 (3)	H19b—C19—O4	110.9 (6)
C25—O6—C24	111.88 (3)	H19b—C19—C20	107.8 (6)
C18—O4—C19	110.71 (3)	H19b—C19—H19a	105.5 (8)
C22—O5—C23	114.10 (3)	C4—C5—C6	118.62 (3)
C15—N4—C14	114.47 (3)	H5—C5—C6	120.6 (6)
C26—N4—C14	119.21 (3)	H5—C5—C4	120.8 (6)
C26—N4—C15	118.54 (3)	C19—C20—N1	114.33 (3)
C6—N2—N3	115.60 (3)	H20a—C20—N1	107.8 (5)
C20—N1—C1	122.38 (3)	H20a—C20—C19	107.9 (5)
C21—N1—C1	116.47 (3)	H20b—C20—N1	108.1 (6)
C21—N1—C20	116.96 (3)	H20b—C20—C19	111.5 (6)
C8—N3—N2	111.81 (3)	H20b—C20—H20a	107.0 (8)
C11—C12—C14	120.91 (3)	C5—C4—C3	120.74 (3)
C13—C12—C14	118.61 (3)	H4—C4—C3	118.4 (6)
C13—C12—C11	120.09 (3)	H4—C4—C5	120.8 (6)
C9—C8—N3	117.87 (3)	C17—C18—O4	108.89 (3)
C13—C8—N3	121.37 (3)	H18a—C18—O4	109.1 (7)
C13—C8—C9	120.66 (3)	H18a—C18—C17	112.1 (7)
C1—C2—C7	118.82 (3)	H18b—C18—O4	109.0 (6)
C3—C2—C7	119.38 (3)	H18b—C18—C17	108.9 (6)
C3—C2—C1	120.91 (3)	H18b—C18—H18a	108.8 (9)
N4—C14—O2	122.10 (3)	C15—C16—O3	113.62 (3)
C12—C14—O2	119.42 (3)	H16a—C16—O3	109.4 (6)
C12—C14—N4	118.21 (3)	H16a—C16—C15	109.8 (6)
C10—C11—C12	119.88 (3)	H16b—C16—O3	106.0 (6)
H11—C11—C12	122.6 (6)	H16b—C16—C15	108.0 (6)
H11—C11—C10	117.4 (6)	H16b—C16—H16a	110.0 (8)
C16—C15—N4	112.94 (3)	C24—C23—O5	107.97 (3)
H15a—C15—N4	107.9 (5)	H23a—C23—O5	109.7 (6)
H15a—C15—C16	108.2 (6)	H23a—C23—C24	109.4 (6)
H15b—C15—N4	111.0 (6)	H23b—C23—O5	111.0 (6)
H15b—C15—C16	107.5 (6)	H23b—C23—C24	109.5 (6)
H15b—C15—H15a	109.3 (8)	H23b—C23—H23a	109.3 (9)
C10—C9—C8	119.23 (3)	C18—C17—O3	105.84 (3)
H9—C9—C8	119.7 (6)	H17a—C17—O3	111.9 (5)
H9—C9—C10	121.1 (6)	H17a—C17—C18	110.6 (6)
C7—C6—N2	112.66 (3)	H17b—C17—O3	110.0 (6)
C5—C6—N2	126.27 (3)	H17b—C17—C18	110.6 (6)
C5—C6—C7	120.97 (3)	H17b—C17—H17a	108.0 (8)
C6—C7—C2	120.01 (3)	C23—C24—O6	107.51 (3)
H7—C7—C2	121.1 (6)	H24a—C24—O6	109.4 (6)
H7—C7—C6	118.7 (6)	H24a—C24—C23	110.6 (6)
C9—C10—C11	120.40 (3)	H24b—C24—O6	111.0 (7)
H10—C10—C11	119.9 (6)	H24b—C24—C23	109.5 (7)
H10—C10—C9	119.7 (6)	H24b—C24—H24a	108.9 (9)
C25—C26—N4	115.36 (3)	C22—C21—N1	114.52 (3)
H26a—C26—N4	109.2 (6)	H21a—C21—N1	108.5 (6)
H26a—C26—C25	108.0 (6)	H21a—C21—C22	109.5 (6)
H26b—C26—N4	108.3 (5)	H21b—C21—N1	108.2 (6)
H26b—C26—C25	107.9 (5)	H21b—C21—C22	107.3 (6)
H26b—C26—H26a	107.9 (8)	H21b—C21—H21a	108.7 (8)
N1—C1—O1	121.96 (3)	C26—C25—O6	111.08 (3)
C2—C1—O1	118.79 (3)	H25a—C25—O6	109.7 (6)
C2—C1—N1	118.98 (3)	H25a—C25—C26	107.9 (6)
C4—C3—C2	119.87 (3)	H25b—C25—O6	109.1 (5)
H3—C3—C2	120.2 (6)	H25b—C25—C26	112.4 (6)
H3—C3—C4	119.9 (6)	H25b—C25—H25a	106.5 (8)
C8—C13—C12	119.64 (3)	C21—C22—O5	113.91 (3)
H13—C13—C12	121.9 (6)	H22a—C22—O5	109.5 (6)
H13—C13—C8	118.5 (6)	H22a—C22—C21	110.4 (6)
C20—C19—O4	110.93 (3)	H22b—C22—O5	104.2 (6)
H19a—C19—O4	109.5 (6)	H22b—C22—C21	108.0 (6)
H19a—C19—C20	112.1 (6)	H22b—C22—H22a	110.7 (9)
			
O3—C16—C15—N4	75.11 (3)	C8—C13—C12—C14	172.35 (3)
O3—C17—C18—O4	−166.08 (3)	C8—C13—C12—C11	−0.50 (4)
O6—C24—C23—O5	−166.56 (3)	C2—C7—C6—C5	6.63 (4)
O6—C25—C26—N4	68.96 (3)	C2—C1—N1—C20	30.84 (4)
O4—C19—C20—N1	73.35 (3)	C2—C1—N1—C21	−172.90 (3)
O5—C22—C21—N1	62.93 (3)	C2—C3—C4—C5	4.13 (4)
O2—C14—N4—C15	3.22 (4)	C14—N4—C15—C16	75.33 (3)
O2—C14—N4—C26	−145.64 (3)	C14—N4—C26—C25	−158.72 (3)
O2—C14—C12—C11	51.03 (4)	C14—C12—C11—C10	−174.68 (3)
O2—C14—C12—C13	−121.76 (3)	C15—N4—C26—C25	53.67 (3)
O1—C1—N1—C20	−155.20 (3)	C15—C16—O3—C17	−92.93 (3)
O1—C1—N1—C21	1.05 (4)	C6—C7—C2—C1	163.55 (3)
O1—C1—C2—C7	−123.76 (4)	C6—C7—C2—C3	−5.77 (4)
O1—C1—C2—C3	45.39 (4)	C6—C5—C4—C3	−3.32 (4)
N4—C14—C12—C11	−134.73 (3)	C7—C2—C3—C4	0.46 (4)
N4—C14—C12—C13	52.47 (3)	C7—C6—C5—C4	−2.05 (4)
N2—N3—C8—C9	−167.33 (3)	C10—C11—C12—C13	−1.99 (4)
N2—N3—C8—C13	16.24 (4)	C10—C9—C8—C13	−3.28 (4)
N2—C6—C7—C2	−169.91 (3)	C26—N4—C15—C16	−135.58 (3)
N2—C6—C5—C4	173.99 (4)	C26—C25—O6—C24	−164.27 (3)
N1—C1—C2—C7	50.39 (4)	C1—N1—C20—C19	−138.24 (3)
N1—C1—C2—C3	−140.47 (3)	C1—N1—C21—C22	73.02 (3)
N3—N2—C6—C7	144.69 (3)	C1—C2—C3—C4	−168.62 (3)
N3—N2—C6—C5	−31.63 (4)	C19—O4—C18—C17	−176.96 (3)
N3—C8—C9—C10	−179.74 (3)	C19—C20—N1—C21	65.61 (3)
N3—C8—C13—C12	179.50 (3)	C20—N1—C21—C22	−129.41 (3)
C12—C14—N4—C15	−170.85 (3)	C20—C19—O4—C18	−169.69 (3)
C12—C14—N4—C26	40.28 (3)	C18—C17—O3—C16	−163.18 (3)
C12—C11—C10—C9	1.87 (4)	C23—O5—C22—C21	−94.39 (3)
C12—C13—C8—C9	3.16 (4)	C23—C24—O6—C25	−161.06 (3)
C8—N3—N2—C6	−171.60 (3)	C24—C23—O5—C22	−160.18 (3)
C8—C9—C10—C11	0.75 (4)		

### Poly[[{µ-7,16-[3,3'-(diazene-1,2-diyl)bis(1,3-phenylenecarbonyl)]-1,4,10,13-\ tetraoxa-7,16-diazacyclooctadecane}bis{tetrakis[3,5-bis(trifluoromethyl)\ phenyl]borato}disodium] benzene disolvate] (2) Crystal data

**Table:**

[Na_2_(C_32_H_12_F_24_B)_2_(C_26_H_32_N_4_O_6_)]·2C_6_H_6_	*Z* = 2
*M**_r_* = 2425.20	*F*(000) = 2440
Triclinic, *P*1	*D*_x_ = 1.583 Mg m^−^^3^
*a* = 13.5857 (6) Å	Cu *K*α radiation, λ = 1.54178 Å
*b* = 13.8054 (6) Å	Cell parameters from 5022 reflections
*c* = 27.8552 (11) Å	θ = 3.2–50.6°
α = 84.152 (2)°	µ = 1.49 mm^−^^1^
β = 78.185 (2)°	*T* = 100 K
γ = 89.238 (2)°	Block, yellow
*V* = 5087.0 (4) Å^3^	0.30 × 0.28 × 0.24 mm

### Poly[[{µ-7,16-[3,3'-(diazene-1,2-diyl)bis(1,3-phenylenecarbonyl)]-1,4,10,13-\ tetraoxa-7,16-diazacyclooctadecane}bis{tetrakis[3,5-bis(trifluoromethyl)\ phenyl]borato}disodium] benzene disolvate] (2) Data collection

**Table:**

Bruker APEXII CCD diffractometer	18230 reflections with *I* > 2σ(*I*)
φ and ω scans	*R*_int_ = 0.044
Absorption correction: multi-scan (SADABS; Krause *et al.*, 2015)	θ_max_ = 75.0°, θ_min_ = 1.6°
*T*_min_ = 0.342, *T*_max_ = 0.471	*h* = −17→16
168897 measured reflections	*k* = −17→17
20857 independent reflections	*l* = −34→34

### Poly[[{µ-7,16-[3,3'-(diazene-1,2-diyl)bis(1,3-phenylenecarbonyl)]-1,4,10,13-\ tetraoxa-7,16-diazacyclooctadecane}bis{tetrakis[3,5-bis(trifluoromethyl)\ phenyl]borato}disodium] benzene disolvate] (2) Refinement

**Table:**

Refinement on *F*^2^	Primary atom site location: dual
Least-squares matrix: full	Hydrogen site location: inferred from neighbouring sites
*R*[*F*^2^ > 2σ(*F*^2^)] = 0.071	H-atom parameters constrained
*wR*(*F*^2^) = 0.205	*w* = 1/[σ^2^(*F*_o_^2^) + (0.107*P*)^2^ + 6.5402*P*] where *P* = (*F*_o_^2^ + 2*F*_c_^2^)/3
*S* = 1.08	(Δ/σ)_max_ = 0.001
20857 reflections	Δρ_max_ = 1.45 e Å^−^^3^
1473 parameters	Δρ_min_ = −0.76 e Å^−^^3^
244 restraints	

### Poly[[{µ-7,16-[3,3'-(diazene-1,2-diyl)bis(1,3-phenylenecarbonyl)]-1,4,10,13-\ tetraoxa-7,16-diazacyclooctadecane}bis{tetrakis[3,5-bis(trifluoromethyl)\ phenyl]borato}disodium] benzene disolvate] (2) Special details

**Table:**

Geometry. All esds (except the esd in the dihedral angle between two l.s. planes) are estimated using the full covariance matrix. The cell esds are taken into account individually in the estimation of esds in distances, angles and torsion angles; correlations between esds in cell parameters are only used when they are defined by crystal symmetry. An approximate (isotropic) treatment of cell esds is used for estimating esds involving l.s. planes.

### Poly[[{µ-7,16-[3,3'-(diazene-1,2-diyl)bis(1,3-phenylenecarbonyl)]-1,4,10,13-\ tetraoxa-7,16-diazacyclooctadecane}bis{tetrakis[3,5-bis(trifluoromethyl)\ phenyl]borato}disodium] benzene disolvate] (2) Fractional atomic coordinates and isotropic or equivalent isotropic displacement parameters (Å^2^)

**Table:**

	*x*	*y*	*z*	*U*_iso_*/*U*_eq_	Occ. (<1)
Na1	0.79144 (8)	0.56214 (8)	0.69720 (4)	0.0363 (2)	
Na2	0.82395 (8)	0.04313 (7)	0.80949 (5)	0.0404 (3)	
F1	0.64788 (12)	0.08277 (12)	0.84767 (7)	0.0397 (4)	
F49	1.16929 (13)	0.95580 (13)	0.33335 (7)	0.0436 (4)	
F42	0.45824 (15)	−0.16014 (13)	0.69901 (7)	0.0464 (4)	
F2	0.67148 (13)	0.12595 (14)	0.77007 (7)	0.0488 (4)	
F32	0.11455 (15)	−0.44091 (12)	0.92366 (8)	0.0496 (5)	
F25	0.17784 (15)	0.28699 (14)	0.87623 (7)	0.0492 (4)	
F28	0.00420 (15)	0.00748 (14)	0.80757 (8)	0.0514 (5)	
F31	0.00300 (15)	−0.43162 (14)	0.87917 (7)	0.0501 (5)	
F27	−0.06317 (15)	−0.11943 (16)	0.79216 (8)	0.0582 (5)	
F48	1.08307 (17)	0.92317 (14)	0.28133 (7)	0.0553 (5)	
F3	0.55899 (14)	0.19707 (12)	0.82154 (9)	0.0540 (5)	
O1	0.73355 (14)	0.92050 (12)	0.79017 (7)	0.0319 (4)	
O6	0.76352 (14)	0.41487 (13)	0.74800 (7)	0.0338 (4)	
F20	0.82181 (15)	0.74482 (16)	0.23338 (7)	0.0537 (5)	
F50	1.02660 (15)	1.02375 (12)	0.33210 (9)	0.0579 (5)	
F30	−0.02954 (15)	−0.38043 (15)	0.95094 (7)	0.0533 (5)	
F26	0.32964 (16)	0.28838 (14)	0.88404 (9)	0.0599 (6)	
F23	0.37819 (14)	0.73798 (19)	0.40928 (7)	0.0614 (6)	
F18	0.77922 (18)	0.89430 (14)	0.23004 (7)	0.0585 (5)	
F17	0.48010 (16)	1.08002 (16)	0.42721 (8)	0.0591 (5)	
F35	0.53559 (15)	−0.46707 (15)	0.91413 (10)	0.0635 (6)	
F19	0.68738 (16)	0.78969 (18)	0.20878 (7)	0.0589 (5)	
F44	0.32652 (15)	−0.07330 (18)	0.70404 (7)	0.0576 (5)	
F46	1.04253 (16)	0.55450 (16)	0.45488 (8)	0.0627 (6)	
O5	0.63713 (14)	0.57436 (13)	0.74975 (7)	0.0340 (4)	
F33	0.39215 (19)	−0.51580 (14)	0.95178 (9)	0.0675 (7)	
F41	0.16972 (17)	−0.02471 (18)	1.08371 (7)	0.0606 (5)	
F40	0.02645 (14)	−0.01428 (16)	1.06328 (9)	0.0634 (6)	
F34	0.4135 (2)	−0.45001 (15)	0.87792 (8)	0.0670 (7)	
F29	0.08372 (15)	−0.07806 (19)	0.75250 (7)	0.0615 (6)	
F43	0.4703 (2)	−0.01010 (15)	0.67082 (7)	0.0620 (6)	
O4	0.84497 (15)	0.71919 (14)	0.70406 (8)	0.0382 (4)	
F24	0.2121 (3)	0.33598 (13)	0.94105 (8)	0.0777 (8)	
F15	0.52199 (18)	1.18410 (17)	0.47126 (8)	0.0646 (6)	
F47	1.17825 (15)	0.62878 (16)	0.42027 (11)	0.0697 (7)	
O3	0.94666 (16)	0.54332 (15)	0.72298 (8)	0.0418 (5)	
F37	0.47979 (18)	−0.1502 (2)	1.05499 (8)	0.0709 (7)	
O2	0.87808 (16)	0.19102 (14)	0.81237 (9)	0.0422 (5)	
F16	0.6039 (2)	1.17583 (17)	0.39756 (8)	0.0681 (6)	
F21	0.37267 (15)	0.8021 (2)	0.33648 (10)	0.0790 (8)	
F39	0.1001 (2)	0.11241 (17)	1.08176 (9)	0.0800 (8)	
F45	1.0975 (2)	0.55171 (16)	0.37743 (9)	0.0705 (6)	
F36	0.3411 (2)	−0.2174 (3)	1.08526 (8)	0.0896 (10)	
N3	0.66167 (16)	0.78773 (15)	0.76902 (8)	0.0287 (4)	
N2	0.77477 (17)	0.55690 (16)	0.89839 (8)	0.0315 (4)	
N1	0.77800 (17)	0.50508 (16)	0.93773 (8)	0.0323 (5)	
F51	0.6968 (3)	0.3585 (2)	0.49322 (13)	0.0542 (7)	0.651 (4)
N4	0.93762 (17)	0.34103 (15)	0.78021 (9)	0.0324 (5)	
F22	0.40159 (19)	0.6514 (2)	0.34986 (11)	0.0832 (8)	
C79	0.41705 (18)	−0.34668 (18)	0.94001 (9)	0.0272 (5)	
C81	0.4380 (2)	−0.44388 (19)	0.92118 (10)	0.0308 (5)	
C61	0.43523 (19)	−0.04553 (18)	0.75695 (9)	0.0290 (5)	
C68	0.14867 (17)	−0.10063 (17)	0.85063 (9)	0.0255 (5)	
H68	0.169181	−0.035850	0.838088	0.031*	
C2	0.68657 (18)	0.79091 (18)	0.85298 (10)	0.0282 (5)	
C53	1.02377 (19)	0.85840 (18)	0.36372 (9)	0.0286 (5)	
C59	0.37658 (17)	−0.06285 (16)	0.84663 (9)	0.0242 (4)	
C71	0.08055 (19)	−0.28456 (18)	0.88820 (9)	0.0283 (5)	
C75	0.35422 (17)	−0.17995 (17)	0.93006 (9)	0.0239 (4)	
C58	1.0760 (2)	0.94004 (19)	0.32896 (10)	0.0318 (5)	
C60	0.36990 (18)	−0.08507 (17)	0.79970 (9)	0.0256 (5)	
H60	0.318774	−0.128952	0.796649	0.031*	
C1	0.69211 (18)	0.84015 (17)	0.80227 (10)	0.0282 (5)	
C74	0.0253 (2)	−0.0837 (2)	0.79684 (11)	0.0363 (6)	
C83	0.25377 (17)	−0.00701 (17)	0.92802 (9)	0.0262 (5)	
C63	0.52154 (19)	0.03838 (17)	0.80627 (10)	0.0300 (5)	
C72	0.16071 (18)	−0.24374 (17)	0.90343 (9)	0.0249 (5)	
H72	0.190336	−0.280016	0.927587	0.030*	
C76	0.37507 (19)	−0.16899 (19)	0.97609 (9)	0.0292 (5)	
H76	0.363200	−0.107774	0.988935	0.035*	
C67	0.19896 (17)	−0.15134 (17)	0.88439 (9)	0.0237 (4)	
C54	1.0797 (2)	0.7798 (2)	0.37699 (10)	0.0343 (5)	
H54	1.151002	0.780183	0.367400	0.041*	
C4	0.73122 (19)	0.65049 (18)	0.90253 (10)	0.0294 (5)	
C64	0.45656 (18)	−0.00113 (17)	0.84866 (10)	0.0275 (5)	
H64	0.466473	0.013996	0.879855	0.033*	
C80	0.38002 (17)	−0.27064 (17)	0.91234 (9)	0.0243 (4)	
H80	0.371781	−0.280302	0.880107	0.029*	
C70	0.03493 (18)	−0.2345 (2)	0.85324 (10)	0.0302 (5)	
H70	−0.018687	−0.262961	0.842227	0.036*	
C36	0.6486 (2)	0.9616 (2)	0.43628 (10)	0.0347 (6)	
H36	0.613006	0.946399	0.411959	0.042*	
C3	0.72610 (19)	0.69805 (18)	0.85692 (9)	0.0287 (5)	
H3	0.749959	0.666705	0.827983	0.034*	
C8	0.8276 (2)	0.41532 (19)	0.92672 (10)	0.0326 (5)	
C51	0.86629 (19)	0.78246 (19)	0.40981 (9)	0.0299 (5)	
C20	0.57063 (19)	0.72539 (17)	0.78492 (10)	0.0284 (5)	
H20A	0.510894	0.767747	0.786614	0.034*	
H20B	0.569927	0.694866	0.818730	0.034*	
C69	0.06970 (18)	−0.14197 (19)	0.83484 (9)	0.0281 (5)	
C52	0.92043 (19)	0.86063 (18)	0.37989 (9)	0.0290 (5)	
H52	0.884826	0.916727	0.370507	0.035*	
C43	0.68772 (19)	0.78214 (18)	0.38127 (9)	0.0289 (5)	
C84	0.2630 (2)	0.08982 (18)	0.90750 (10)	0.0292 (5)	
H84	0.297484	0.103761	0.874252	0.035*	
C85	0.2235 (2)	0.16704 (19)	0.93402 (10)	0.0325 (5)	
C35	0.7185 (2)	0.8954 (2)	0.45012 (10)	0.0349 (6)	
F6	0.7269 (3)	0.6070 (4)	0.62560 (10)	0.1230 (15)	
C89	0.20010 (19)	−0.02175 (18)	0.97705 (10)	0.0311 (5)	
H89	0.188559	−0.086555	0.992176	0.037*	
C6	0.6597 (2)	0.7883 (2)	0.94151 (11)	0.0367 (6)	
H6	0.637177	0.819884	0.970511	0.044*	
C86	0.1739 (2)	0.1504 (2)	0.98276 (11)	0.0358 (6)	
H86	0.147809	0.202850	1.000973	0.043*	
C65	0.5989 (2)	0.1104 (2)	0.81109 (12)	0.0382 (6)	
C45	0.6853 (2)	0.78828 (18)	0.29371 (9)	0.0292 (5)	
C44	0.73653 (19)	0.79285 (18)	0.33199 (9)	0.0284 (5)	
H44	0.807184	0.803648	0.324136	0.034*	
C78	0.4328 (2)	−0.3348 (2)	0.98695 (10)	0.0329 (5)	
H78	0.456530	−0.386792	1.006505	0.040*	
C5	0.6980 (2)	0.6952 (2)	0.94536 (10)	0.0346 (6)	
H5	0.701451	0.662418	0.976647	0.042*	
C26	1.0049 (2)	0.42298 (18)	0.78141 (11)	0.0321 (5)	
H26A	1.066804	0.417159	0.756043	0.039*	
H26B	1.024533	0.416920	0.813898	0.039*	
C62	0.51167 (19)	0.01764 (18)	0.75991 (10)	0.0312 (5)	
H62	0.555872	0.045784	0.731059	0.037*	
C57	0.9240 (2)	0.7025 (2)	0.42114 (11)	0.0365 (6)	
H57	0.891073	0.647288	0.440724	0.044*	
C77	0.4128 (2)	−0.2447 (2)	1.00401 (10)	0.0344 (6)	
C47	0.5323 (2)	0.76036 (19)	0.35215 (10)	0.0325 (5)	
C30	0.70528 (19)	0.6976 (2)	0.46927 (9)	0.0314 (5)	
C46	0.5828 (2)	0.77201 (18)	0.30334 (9)	0.0303 (5)	
H46	0.547871	0.768864	0.277268	0.036*	
C48	0.5834 (2)	0.7654 (2)	0.39029 (9)	0.0323 (5)	
H48	0.546763	0.757261	0.423383	0.039*	
C10	0.8990 (2)	0.30204 (18)	0.86919 (11)	0.0334 (6)	
C19	0.5598 (2)	0.64553 (18)	0.75285 (10)	0.0314 (5)	
H19A	0.494271	0.612310	0.765914	0.038*	
H19B	0.558770	0.675754	0.719164	0.038*	
C7	0.6535 (2)	0.83700 (19)	0.89552 (10)	0.0329 (5)	
H7	0.627036	0.900962	0.893397	0.040*	
C21	0.6949 (2)	0.82243 (19)	0.71667 (10)	0.0337 (6)	
H21A	0.673319	0.890718	0.711117	0.040*	
H21B	0.661853	0.782724	0.696763	0.040*	
C49	0.4219 (2)	0.7403 (3)	0.36258 (11)	0.0449 (7)	
C66	0.4225 (2)	−0.0715 (2)	0.70775 (10)	0.0349 (6)	
C9	0.8428 (2)	0.38435 (18)	0.87982 (10)	0.0320 (5)	
H9	0.814519	0.419865	0.854962	0.038*	
C11	0.9078 (2)	0.27262 (18)	0.81853 (12)	0.0348 (6)	
C37	0.6290 (2)	1.0498 (2)	0.45701 (11)	0.0385 (6)	
C16	0.8056 (2)	0.3240 (2)	0.73360 (13)	0.0410 (7)	
H16A	0.793154	0.314045	0.700646	0.049*	
H16B	0.772690	0.270052	0.757355	0.049*	
C18	0.6246 (2)	0.50050 (19)	0.79052 (11)	0.0368 (6)	
H18A	0.552907	0.495019	0.807166	0.044*	
H18B	0.664006	0.517717	0.814712	0.044*	
C73	0.0424 (2)	−0.3839 (2)	0.91049 (11)	0.0378 (6)	
C50	0.7429 (2)	0.8037 (2)	0.24191 (10)	0.0359 (6)	
C88	0.1148 (3)	0.0337 (2)	1.05892 (15)	0.0536 (9)	
C87	0.1632 (2)	0.0548 (2)	1.00431 (11)	0.0369 (6)	
C22	0.8074 (2)	0.81712 (19)	0.69938 (10)	0.0345 (6)	
H22A	0.825618	0.844250	0.664403	0.041*	
H22B	0.840375	0.857922	0.718844	0.041*	
C25	0.9619 (2)	0.52419 (19)	0.77284 (11)	0.0359 (6)	
H25A	0.896995	0.529529	0.796219	0.043*	
H25B	1.008672	0.573517	0.779153	0.043*	
C31	0.6931 (2)	0.6057 (2)	0.45463 (10)	0.0376 (6)	
H31	0.703461	0.598392	0.420412	0.045*	
C29	0.6877 (2)	0.7033 (2)	0.52002 (10)	0.0376 (6)	
H29	0.693023	0.764665	0.531971	0.045*	
C90	0.2362 (3)	0.2692 (2)	0.90921 (11)	0.0418 (7)	
C12	0.9377 (2)	0.2480 (2)	0.90618 (13)	0.0420 (7)	
H12	0.975627	0.190925	0.899268	0.050*	
C14	0.8653 (2)	0.3616 (2)	0.96365 (11)	0.0394 (6)	
H14	0.853714	0.381750	0.995942	0.047*	
F7	0.6254 (5)	0.7164 (3)	0.61997 (10)	0.157 (2)	
C82	0.4279 (3)	−0.2290 (2)	1.05449 (12)	0.0476 (7)	
C55	1.0282 (2)	0.7002 (2)	0.40485 (11)	0.0377 (6)	
C15	0.9177 (2)	0.3234 (2)	0.73211 (11)	0.0377 (6)	
H15A	0.945731	0.259641	0.723084	0.045*	
H15B	0.951147	0.374493	0.706694	0.045*	
C32	0.6664 (3)	0.5241 (2)	0.48841 (13)	0.0472 (7)	
C17	0.6603 (2)	0.4053 (2)	0.77174 (12)	0.0402 (6)	
H17A	0.652136	0.353309	0.799502	0.048*	
H17B	0.619905	0.387419	0.748121	0.048*	
F53	0.6487 (5)	0.4236 (3)	0.42838 (16)	0.0829 (13)	0.651 (4)
C28	0.6624 (2)	0.6211 (3)	0.55363 (11)	0.0450 (7)	
F38	0.4716 (4)	−0.3013 (3)	1.07532 (13)	0.158 (2)	
C13	0.9202 (3)	0.2783 (2)	0.95306 (13)	0.0457 (7)	
H13	0.946168	0.241495	0.978327	0.055*	
C56	1.0863 (2)	0.6095 (2)	0.41498 (12)	0.0455 (7)	
C23	0.9498 (2)	0.7195 (2)	0.70751 (14)	0.0477 (7)	
H23A	0.955118	0.730143	0.741505	0.057*	
H23B	0.985297	0.773788	0.684886	0.057*	
C40	0.7687 (3)	0.9237 (3)	0.48562 (14)	0.0531 (8)	
H40	0.819903	0.882838	0.494885	0.064*	
C24	0.9990 (2)	0.6251 (3)	0.69449 (13)	0.0487 (7)	
H24A	1.002085	0.619156	0.659099	0.058*	
H24B	1.068856	0.625505	0.699831	0.058*	
C33	0.6520 (2)	0.5314 (3)	0.53834 (12)	0.0497 (8)	
H33	0.635388	0.475655	0.561496	0.060*	
B2	0.2969 (2)	−0.10015 (19)	0.89780 (10)	0.0235 (5)	
F8	0.5764 (4)	0.5769 (4)	0.63442 (11)	0.156 (2)	
C42	0.5589 (3)	1.1218 (2)	0.43876 (12)	0.0450 (7)	
C38	0.6770 (3)	1.0742 (3)	0.49333 (15)	0.0531 (8)	
H38	0.662821	1.133556	0.507804	0.064*	
C34	0.6474 (4)	0.4295 (3)	0.47183 (17)	0.0710 (10)	
C27	0.6470 (3)	0.6313 (4)	0.60749 (12)	0.0631 (11)	
C39	0.7463 (3)	1.0095 (3)	0.50793 (18)	0.0667 (11)	
B1	0.7436 (2)	0.7898 (2)	0.42783 (10)	0.0297 (6)	
F4	0.5481 (3)	0.3968 (3)	0.49816 (19)	0.0848 (12)	0.651 (4)
F12	0.7703 (18)	0.9965 (14)	0.5882 (7)	0.1334 (19)	0.229 (6)
C95	0.3063 (3)	−0.5185 (2)	0.76995 (14)	0.0929 (18)	
H95	0.314583	−0.581385	0.785835	0.111*	
C94	0.3364 (3)	−0.5005 (2)	0.71906 (13)	0.0891 (16)	
H94	0.365336	−0.551079	0.700165	0.107*	
C93	0.3243 (2)	−0.4084 (2)	0.69582 (8)	0.0754 (13)	
H93	0.344936	−0.396091	0.661039	0.090*	
C92	0.2821 (2)	−0.33433 (18)	0.72347 (12)	0.0698 (12)	
H92	0.273783	−0.271406	0.707583	0.084*	
C91	0.2519 (2)	−0.3523 (2)	0.77436 (12)	0.0774 (14)	
H91	0.223029	−0.301709	0.793253	0.093*	
C96	0.2640 (2)	−0.4444 (3)	0.79760 (8)	0.0839 (16)	
H96	0.243429	−0.456698	0.832380	0.101*	
C98	0.9851 (4)	0.6908 (3)	0.55626 (16)	0.112 (2)	
H98	0.945941	0.643174	0.546205	0.134*	
C99	1.0817 (4)	0.6686 (3)	0.56317 (19)	0.124 (3)	
H99	1.108459	0.605739	0.557840	0.149*	
C100	1.1391 (3)	0.7383 (4)	0.5779 (2)	0.149 (4)	
H100	1.205034	0.723073	0.582597	0.179*	
C101	1.0999 (4)	0.8302 (3)	0.58567 (19)	0.127 (3)	
H101	1.139092	0.877842	0.595719	0.152*	
C102	1.0033 (4)	0.8524 (3)	0.57876 (19)	0.142 (3)	
H102	0.976575	0.915278	0.584084	0.171*	
C97	0.9460 (4)	0.7827 (4)	0.56405 (17)	0.131 (3)	
H97	0.879998	0.797946	0.559327	0.158*	
C41	0.8018 (5)	1.0322 (4)	0.5480 (3)	0.1022 (18)	
F11	0.7722 (6)	0.9573 (4)	0.5871 (2)	0.1334 (19)	0.771 (6)
F14	0.7752 (5)	1.1196 (4)	0.5645 (2)	0.1277 (17)	0.771 (6)
F5	0.6600 (7)	0.3428 (6)	0.4916 (4)	0.0848 (12)	0.349 (4)
F54	0.5776 (8)	0.4334 (6)	0.4431 (3)	0.0829 (13)	0.349 (4)
F52	0.7243 (6)	0.4212 (4)	0.4222 (2)	0.0542 (7)	0.349 (4)
F13	0.8040 (17)	1.0874 (12)	0.5621 (8)	0.1277 (17)	0.229 (6)
F10	0.8627 (12)	0.9582 (11)	0.5646 (8)	0.1187 (17)	0.229 (6)
F9	0.9024 (4)	1.0369 (3)	0.5308 (2)	0.1187 (17)	0.771 (6)

### Poly[[{µ-7,16-[3,3'-(diazene-1,2-diyl)bis(1,3-phenylenecarbonyl)]-1,4,10,13-\ tetraoxa-7,16-diazacyclooctadecane}bis{tetrakis[3,5-bis(trifluoromethyl)\ phenyl]borato}disodium] benzene disolvate] (2) Atomic displacement parameters (Å^2^)

**Table:**

	*U*^11^	*U*^22^	*U*^33^	*U*^12^	*U*^13^	*U*^23^
Na1	0.0347 (5)	0.0317 (5)	0.0435 (6)	−0.0021 (4)	−0.0130 (5)	0.0012 (4)
Na2	0.0292 (5)	0.0224 (5)	0.0718 (8)	−0.0030 (4)	−0.0142 (5)	−0.0080 (5)
F1	0.0319 (8)	0.0366 (8)	0.0533 (10)	−0.0072 (6)	−0.0105 (7)	−0.0128 (7)
F49	0.0354 (8)	0.0477 (10)	0.0474 (10)	−0.0170 (7)	−0.0151 (7)	0.0118 (8)
F42	0.0630 (11)	0.0385 (9)	0.0442 (9)	0.0117 (8)	−0.0224 (8)	−0.0134 (7)
F2	0.0393 (9)	0.0456 (10)	0.0560 (11)	−0.0181 (8)	0.0000 (8)	0.0034 (8)
F32	0.0565 (11)	0.0286 (8)	0.0664 (12)	−0.0124 (7)	−0.0241 (9)	0.0070 (8)
F25	0.0570 (11)	0.0467 (10)	0.0430 (10)	0.0116 (8)	−0.0132 (8)	0.0049 (8)
F28	0.0608 (12)	0.0443 (10)	0.0578 (11)	0.0163 (8)	−0.0337 (9)	−0.0043 (8)
F31	0.0609 (11)	0.0441 (10)	0.0496 (10)	−0.0276 (9)	−0.0193 (9)	−0.0052 (8)
F27	0.0435 (10)	0.0704 (13)	0.0709 (13)	−0.0032 (9)	−0.0393 (10)	0.0020 (10)
F48	0.0822 (14)	0.0503 (11)	0.0342 (9)	−0.0325 (10)	−0.0177 (9)	0.0076 (8)
F3	0.0441 (10)	0.0222 (8)	0.0938 (15)	−0.0051 (7)	−0.0090 (10)	−0.0066 (8)
O1	0.0323 (9)	0.0206 (8)	0.0440 (10)	−0.0040 (7)	−0.0105 (8)	−0.0023 (7)
O6	0.0348 (9)	0.0212 (8)	0.0473 (11)	−0.0018 (7)	−0.0113 (8)	−0.0053 (7)
F20	0.0568 (11)	0.0655 (12)	0.0335 (9)	0.0280 (10)	0.0002 (8)	−0.0028 (8)
F50	0.0458 (10)	0.0243 (8)	0.0939 (16)	−0.0043 (7)	0.0027 (10)	0.0065 (9)
F30	0.0537 (11)	0.0582 (11)	0.0422 (10)	−0.0246 (9)	0.0023 (8)	0.0009 (8)
F26	0.0592 (12)	0.0326 (9)	0.0894 (16)	−0.0138 (8)	−0.0263 (11)	0.0109 (9)
F23	0.0355 (9)	0.1041 (18)	0.0407 (10)	−0.0165 (10)	−0.0051 (8)	0.0078 (10)
F18	0.0777 (14)	0.0439 (10)	0.0410 (10)	−0.0065 (9)	0.0110 (9)	0.0109 (8)
F17	0.0549 (12)	0.0632 (13)	0.0646 (13)	0.0194 (10)	−0.0231 (10)	−0.0129 (10)
F35	0.0373 (10)	0.0493 (11)	0.1088 (18)	0.0093 (8)	−0.0149 (10)	−0.0328 (12)
F19	0.0567 (12)	0.0943 (16)	0.0271 (8)	0.0073 (11)	−0.0114 (8)	−0.0084 (9)
F44	0.0459 (10)	0.0940 (16)	0.0408 (10)	0.0187 (10)	−0.0219 (8)	−0.0197 (10)
F46	0.0581 (12)	0.0528 (12)	0.0641 (13)	0.0078 (9)	−0.0005 (10)	0.0308 (10)
O5	0.0355 (10)	0.0227 (8)	0.0423 (10)	−0.0020 (7)	−0.0053 (8)	−0.0005 (7)
F33	0.0890 (16)	0.0311 (9)	0.0663 (13)	−0.0153 (10)	0.0221 (12)	−0.0041 (9)
F41	0.0619 (13)	0.0806 (15)	0.0368 (10)	−0.0031 (11)	−0.0048 (9)	−0.0037 (9)
F40	0.0362 (10)	0.0606 (12)	0.0803 (15)	−0.0075 (9)	0.0040 (9)	0.0232 (11)
F34	0.1185 (19)	0.0427 (10)	0.0566 (12)	0.0306 (11)	−0.0504 (13)	−0.0224 (9)
F29	0.0507 (11)	0.0998 (17)	0.0314 (9)	0.0251 (11)	−0.0108 (8)	0.0071 (10)
F43	0.1007 (17)	0.0529 (11)	0.0276 (9)	−0.0200 (11)	−0.0051 (9)	0.0047 (8)
O4	0.0399 (10)	0.0286 (9)	0.0454 (11)	−0.0092 (8)	−0.0112 (9)	0.0053 (8)
F24	0.167 (3)	0.0241 (9)	0.0442 (11)	0.0094 (12)	−0.0242 (13)	−0.0093 (8)
F15	0.0775 (15)	0.0651 (13)	0.0573 (12)	0.0339 (11)	−0.0201 (11)	−0.0264 (10)
F47	0.0399 (10)	0.0562 (12)	0.113 (2)	0.0003 (9)	−0.0306 (11)	0.0239 (12)
O3	0.0413 (11)	0.0367 (10)	0.0479 (12)	−0.0037 (8)	−0.0162 (9)	0.0086 (9)
F37	0.0725 (14)	0.1047 (19)	0.0419 (11)	−0.0375 (13)	−0.0179 (10)	−0.0199 (11)
O2	0.0418 (11)	0.0222 (9)	0.0650 (14)	−0.0025 (8)	−0.0145 (10)	−0.0081 (9)
F16	0.0863 (16)	0.0577 (13)	0.0524 (12)	0.0106 (11)	−0.0050 (11)	0.0116 (10)
F21	0.0367 (10)	0.113 (2)	0.0779 (16)	−0.0099 (11)	−0.0192 (10)	0.0515 (15)
F39	0.110 (2)	0.0496 (12)	0.0602 (14)	−0.0120 (12)	0.0351 (13)	−0.0162 (10)
F45	0.0969 (18)	0.0500 (12)	0.0663 (14)	0.0244 (12)	−0.0222 (13)	−0.0060 (10)
F36	0.0874 (17)	0.147 (3)	0.0331 (10)	−0.0573 (18)	0.0037 (11)	−0.0282 (13)
N3	0.0328 (11)	0.0223 (9)	0.0321 (11)	−0.0056 (8)	−0.0111 (9)	0.0015 (8)
N2	0.0362 (11)	0.0294 (11)	0.0293 (11)	0.0003 (9)	−0.0101 (9)	0.0013 (8)
N1	0.0351 (11)	0.0304 (11)	0.0307 (11)	−0.0056 (9)	−0.0082 (9)	0.0039 (9)
F51	0.078 (2)	0.0340 (13)	0.0499 (14)	−0.0099 (13)	−0.0139 (14)	0.0019 (11)
N4	0.0350 (11)	0.0227 (10)	0.0424 (12)	−0.0007 (8)	−0.0133 (9)	−0.0067 (9)
F22	0.0622 (14)	0.0873 (18)	0.101 (2)	−0.0401 (13)	−0.0126 (13)	−0.0176 (15)
C79	0.0274 (11)	0.0264 (11)	0.0273 (12)	−0.0002 (9)	−0.0068 (9)	0.0018 (9)
C81	0.0343 (13)	0.0274 (12)	0.0307 (12)	0.0011 (10)	−0.0092 (10)	0.0030 (10)
C61	0.0321 (12)	0.0252 (11)	0.0292 (12)	0.0027 (9)	−0.0071 (10)	0.0014 (9)
C68	0.0240 (11)	0.0262 (11)	0.0269 (11)	0.0000 (9)	−0.0075 (9)	−0.0010 (9)
C2	0.0269 (11)	0.0255 (11)	0.0339 (13)	−0.0040 (9)	−0.0103 (10)	−0.0030 (9)
C53	0.0321 (12)	0.0267 (12)	0.0282 (12)	−0.0055 (9)	−0.0091 (10)	−0.0016 (9)
C59	0.0241 (11)	0.0189 (10)	0.0300 (12)	0.0012 (8)	−0.0088 (9)	0.0016 (8)
C71	0.0269 (11)	0.0291 (12)	0.0296 (12)	−0.0051 (9)	−0.0054 (9)	−0.0060 (10)
C75	0.0216 (10)	0.0261 (11)	0.0244 (11)	−0.0032 (8)	−0.0062 (8)	−0.0007 (9)
C58	0.0339 (13)	0.0261 (12)	0.0367 (13)	−0.0051 (10)	−0.0108 (11)	−0.0014 (10)
C60	0.0250 (11)	0.0220 (10)	0.0304 (12)	−0.0002 (8)	−0.0081 (9)	−0.0005 (9)
C1	0.0274 (11)	0.0215 (11)	0.0366 (13)	−0.0007 (9)	−0.0093 (10)	−0.0022 (9)
C74	0.0296 (13)	0.0463 (15)	0.0363 (14)	0.0033 (11)	−0.0142 (11)	−0.0055 (12)
C83	0.0220 (11)	0.0252 (11)	0.0341 (12)	−0.0016 (8)	−0.0114 (9)	−0.0042 (9)
C63	0.0274 (12)	0.0217 (11)	0.0408 (14)	−0.0011 (9)	−0.0085 (10)	0.0011 (10)
C72	0.0252 (11)	0.0266 (11)	0.0237 (11)	−0.0026 (9)	−0.0073 (9)	−0.0015 (9)
C76	0.0293 (12)	0.0324 (12)	0.0285 (12)	0.0007 (10)	−0.0103 (10)	−0.0062 (10)
C67	0.0235 (11)	0.0242 (11)	0.0243 (11)	−0.0008 (8)	−0.0062 (9)	−0.0039 (8)
C54	0.0274 (12)	0.0360 (14)	0.0387 (14)	−0.0034 (10)	−0.0073 (10)	0.0018 (11)
C4	0.0296 (12)	0.0285 (12)	0.0314 (12)	−0.0017 (9)	−0.0091 (10)	−0.0031 (10)
C64	0.0269 (11)	0.0235 (11)	0.0333 (12)	−0.0010 (9)	−0.0105 (10)	−0.0004 (9)
C80	0.0248 (11)	0.0257 (11)	0.0231 (11)	−0.0010 (9)	−0.0072 (9)	−0.0009 (9)
C70	0.0243 (11)	0.0377 (13)	0.0318 (12)	−0.0025 (10)	−0.0093 (9)	−0.0103 (10)
C36	0.0357 (13)	0.0401 (14)	0.0267 (12)	−0.0006 (11)	−0.0035 (10)	−0.0019 (10)
C3	0.0311 (12)	0.0263 (11)	0.0298 (12)	−0.0007 (9)	−0.0083 (10)	−0.0039 (9)
C8	0.0326 (13)	0.0275 (12)	0.0370 (13)	−0.0081 (10)	−0.0096 (10)	0.0064 (10)
C51	0.0315 (12)	0.0315 (12)	0.0272 (12)	−0.0038 (10)	−0.0075 (10)	−0.0018 (9)
C20	0.0286 (12)	0.0236 (11)	0.0341 (12)	−0.0027 (9)	−0.0093 (10)	−0.0016 (9)
C69	0.0240 (11)	0.0352 (13)	0.0270 (12)	0.0038 (9)	−0.0078 (9)	−0.0072 (10)
C52	0.0327 (12)	0.0271 (12)	0.0285 (12)	−0.0020 (9)	−0.0096 (10)	−0.0019 (9)
C43	0.0324 (12)	0.0286 (12)	0.0251 (12)	−0.0015 (9)	−0.0056 (9)	0.0004 (9)
C84	0.0341 (13)	0.0253 (11)	0.0315 (12)	0.0008 (9)	−0.0138 (10)	−0.0048 (9)
C85	0.0391 (14)	0.0255 (12)	0.0374 (14)	0.0012 (10)	−0.0170 (11)	−0.0055 (10)
C35	0.0334 (13)	0.0387 (14)	0.0326 (13)	−0.0010 (11)	−0.0060 (11)	−0.0041 (11)
F6	0.105 (2)	0.231 (4)	0.0418 (13)	0.066 (3)	−0.0362 (14)	−0.0198 (19)
C89	0.0268 (12)	0.0243 (11)	0.0406 (14)	−0.0025 (9)	−0.0024 (10)	−0.0045 (10)
C6	0.0387 (14)	0.0393 (14)	0.0340 (14)	−0.0001 (11)	−0.0078 (11)	−0.0118 (11)
C86	0.0337 (13)	0.0278 (12)	0.0472 (16)	0.0014 (10)	−0.0070 (11)	−0.0115 (11)
C65	0.0318 (13)	0.0257 (12)	0.0544 (17)	−0.0046 (10)	−0.0030 (12)	−0.0025 (11)
C45	0.0387 (13)	0.0233 (11)	0.0250 (12)	0.0023 (9)	−0.0067 (10)	0.0002 (9)
C44	0.0308 (12)	0.0260 (11)	0.0277 (12)	−0.0014 (9)	−0.0055 (10)	0.0002 (9)
C78	0.0375 (14)	0.0343 (13)	0.0286 (12)	0.0035 (10)	−0.0132 (10)	0.0020 (10)
C5	0.0373 (14)	0.0388 (14)	0.0295 (13)	−0.0049 (11)	−0.0112 (11)	−0.0016 (10)
C26	0.0323 (13)	0.0245 (12)	0.0419 (14)	−0.0039 (10)	−0.0142 (11)	−0.0004 (10)
C62	0.0305 (12)	0.0231 (11)	0.0369 (13)	0.0002 (9)	−0.0034 (10)	0.0047 (10)
C57	0.0333 (13)	0.0349 (14)	0.0387 (14)	−0.0059 (11)	−0.0070 (11)	0.0085 (11)
C77	0.0382 (14)	0.0407 (14)	0.0277 (13)	0.0021 (11)	−0.0147 (11)	−0.0036 (11)
C47	0.0347 (13)	0.0320 (13)	0.0304 (13)	−0.0057 (10)	−0.0097 (10)	0.0052 (10)
C30	0.0249 (11)	0.0433 (14)	0.0254 (12)	−0.0009 (10)	−0.0052 (9)	0.0004 (10)
C46	0.0411 (14)	0.0251 (11)	0.0264 (12)	−0.0020 (10)	−0.0124 (10)	0.0016 (9)
C48	0.0328 (13)	0.0387 (14)	0.0239 (11)	−0.0041 (10)	−0.0056 (10)	0.0041 (10)
C10	0.0322 (13)	0.0215 (11)	0.0469 (15)	−0.0058 (9)	−0.0116 (11)	0.0028 (10)
C19	0.0307 (12)	0.0274 (12)	0.0379 (13)	−0.0039 (10)	−0.0111 (10)	−0.0036 (10)
C7	0.0318 (13)	0.0294 (12)	0.0401 (14)	0.0004 (10)	−0.0110 (11)	−0.0078 (10)
C21	0.0425 (14)	0.0270 (12)	0.0333 (13)	−0.0084 (10)	−0.0148 (11)	0.0040 (10)
C49	0.0385 (15)	0.0590 (19)	0.0363 (15)	−0.0132 (13)	−0.0134 (12)	0.0125 (13)
C66	0.0378 (14)	0.0346 (13)	0.0312 (13)	0.0033 (11)	−0.0070 (11)	0.0013 (10)
C9	0.0341 (13)	0.0245 (11)	0.0382 (14)	−0.0046 (10)	−0.0128 (11)	0.0041 (10)
C11	0.0307 (13)	0.0209 (11)	0.0545 (17)	−0.0011 (9)	−0.0135 (12)	−0.0015 (11)
C37	0.0383 (14)	0.0396 (15)	0.0356 (14)	0.0023 (12)	−0.0029 (11)	−0.0044 (11)
C16	0.0446 (16)	0.0270 (13)	0.0586 (18)	0.0024 (11)	−0.0217 (14)	−0.0153 (12)
C18	0.0423 (15)	0.0268 (12)	0.0397 (14)	−0.0022 (11)	−0.0065 (12)	0.0004 (11)
C73	0.0375 (14)	0.0378 (14)	0.0394 (15)	−0.0152 (11)	−0.0110 (12)	−0.0010 (11)
C50	0.0432 (15)	0.0371 (14)	0.0264 (12)	0.0059 (11)	−0.0068 (11)	−0.0002 (10)
C88	0.0452 (17)	0.0307 (14)	0.072 (2)	−0.0054 (13)	0.0226 (16)	−0.0109 (14)
C87	0.0320 (13)	0.0311 (13)	0.0453 (15)	−0.0040 (10)	−0.0003 (11)	−0.0083 (11)
C22	0.0448 (15)	0.0241 (12)	0.0330 (13)	−0.0095 (10)	−0.0071 (11)	0.0044 (10)
C25	0.0427 (15)	0.0236 (12)	0.0426 (15)	−0.0032 (10)	−0.0122 (12)	−0.0020 (10)
C31	0.0424 (15)	0.0412 (15)	0.0301 (13)	−0.0076 (12)	−0.0126 (11)	0.0032 (11)
C29	0.0356 (14)	0.0502 (16)	0.0267 (13)	0.0074 (12)	−0.0073 (10)	−0.0017 (11)
C90	0.0601 (19)	0.0273 (13)	0.0413 (15)	0.0047 (12)	−0.0166 (14)	−0.0072 (11)
C12	0.0409 (15)	0.0247 (12)	0.0615 (19)	−0.0018 (11)	−0.0184 (14)	0.0078 (12)
C14	0.0454 (16)	0.0355 (14)	0.0376 (14)	−0.0068 (12)	−0.0154 (12)	0.0101 (11)
F7	0.301 (6)	0.137 (3)	0.0295 (12)	0.101 (4)	−0.030 (2)	−0.0163 (16)
C82	0.066 (2)	0.0480 (17)	0.0356 (15)	0.0058 (15)	−0.0245 (15)	−0.0087 (13)
C55	0.0350 (14)	0.0366 (14)	0.0399 (15)	−0.0013 (11)	−0.0091 (11)	0.0078 (11)
C15	0.0429 (15)	0.0299 (13)	0.0446 (15)	0.0033 (11)	−0.0144 (12)	−0.0133 (11)
C32	0.0481 (17)	0.0437 (17)	0.0520 (18)	−0.0107 (13)	−0.0215 (14)	0.0089 (14)
C17	0.0392 (15)	0.0243 (12)	0.0558 (18)	−0.0051 (11)	−0.0072 (13)	−0.0027 (12)
F53	0.130 (4)	0.0579 (18)	0.070 (2)	0.007 (3)	−0.038 (3)	−0.0160 (17)
C28	0.0383 (15)	0.063 (2)	0.0302 (14)	0.0057 (14)	−0.0070 (11)	0.0091 (13)
F38	0.321 (6)	0.110 (2)	0.101 (2)	0.120 (3)	−0.157 (3)	−0.061 (2)
C13	0.0509 (18)	0.0372 (15)	0.0500 (18)	−0.0011 (13)	−0.0207 (14)	0.0115 (13)
C56	0.0415 (16)	0.0458 (17)	0.0455 (17)	−0.0017 (13)	−0.0081 (13)	0.0115 (13)
C23	0.0387 (15)	0.0366 (15)	0.066 (2)	−0.0116 (12)	−0.0121 (14)	0.0050 (14)
C40	0.0515 (19)	0.0541 (19)	0.065 (2)	0.0148 (15)	−0.0293 (17)	−0.0251 (17)
C24	0.0390 (16)	0.0509 (18)	0.0539 (19)	−0.0041 (13)	−0.0101 (14)	0.0081 (15)
C33	0.0399 (16)	0.062 (2)	0.0433 (17)	−0.0098 (14)	−0.0139 (13)	0.0234 (15)
B2	0.0253 (12)	0.0212 (11)	0.0258 (12)	−0.0022 (9)	−0.0101 (10)	−0.0002 (9)
F8	0.159 (4)	0.245 (6)	0.0458 (16)	−0.094 (4)	0.0219 (19)	−0.001 (2)
C42	0.0503 (17)	0.0460 (17)	0.0369 (15)	0.0096 (14)	−0.0047 (13)	−0.0064 (12)
C38	0.056 (2)	0.0452 (18)	0.066 (2)	0.0073 (15)	−0.0213 (17)	−0.0229 (16)
C34	0.088 (3)	0.0485 (19)	0.087 (3)	−0.0143 (18)	−0.043 (2)	−0.0001 (18)
C27	0.066 (2)	0.088 (3)	0.0286 (16)	0.018 (2)	−0.0040 (15)	0.0117 (17)
C39	0.070 (2)	0.063 (2)	0.085 (3)	0.0181 (19)	−0.042 (2)	−0.039 (2)
B1	0.0301 (14)	0.0340 (14)	0.0247 (13)	−0.0012 (11)	−0.0058 (10)	−0.0018 (11)
F4	0.072 (2)	0.0543 (19)	0.130 (3)	−0.0187 (16)	−0.024 (2)	−0.0120 (19)
F12	0.213 (4)	0.114 (4)	0.117 (3)	0.029 (4)	−0.118 (3)	−0.050 (3)
C95	0.096 (4)	0.069 (3)	0.116 (5)	−0.036 (3)	−0.044 (3)	0.033 (3)
C94	0.103 (4)	0.066 (3)	0.107 (4)	0.003 (3)	−0.024 (3)	−0.046 (3)
C93	0.075 (3)	0.105 (4)	0.044 (2)	−0.019 (3)	−0.0058 (19)	−0.010 (2)
C92	0.054 (2)	0.054 (2)	0.103 (4)	−0.0120 (17)	−0.023 (2)	0.001 (2)
C91	0.045 (2)	0.101 (4)	0.089 (3)	−0.009 (2)	−0.001 (2)	−0.048 (3)
C96	0.059 (3)	0.138 (5)	0.052 (2)	−0.039 (3)	−0.0033 (19)	−0.007 (3)
C98	0.147 (6)	0.111 (5)	0.070 (3)	0.021 (4)	−0.007 (4)	−0.006 (3)
C99	0.096 (5)	0.096 (4)	0.153 (7)	−0.009 (4)	0.046 (5)	−0.032 (4)
C100	0.077 (4)	0.117 (6)	0.225 (10)	−0.012 (4)	0.053 (5)	−0.049 (6)
C101	0.114 (5)	0.119 (5)	0.124 (6)	−0.002 (4)	0.043 (5)	−0.040 (5)
C102	0.186 (9)	0.152 (7)	0.102 (5)	0.063 (7)	−0.043 (6)	−0.056 (5)
C97	0.195 (9)	0.134 (6)	0.077 (4)	0.050 (6)	−0.048 (5)	−0.031 (4)
C41	0.133 (4)	0.071 (3)	0.137 (4)	0.053 (3)	−0.084 (4)	−0.067 (3)
F11	0.213 (4)	0.114 (4)	0.117 (3)	0.029 (4)	−0.118 (3)	−0.050 (3)
F14	0.180 (4)	0.087 (3)	0.169 (3)	0.067 (3)	−0.126 (3)	−0.092 (3)
F5	0.072 (2)	0.0543 (19)	0.130 (3)	−0.0187 (16)	−0.024 (2)	−0.0120 (19)
F54	0.130 (4)	0.0579 (18)	0.070 (2)	0.007 (3)	−0.038 (3)	−0.0160 (17)
F52	0.078 (2)	0.0340 (13)	0.0499 (14)	−0.0099 (13)	−0.0139 (14)	0.0019 (11)
F13	0.180 (4)	0.087 (3)	0.169 (3)	0.067 (3)	−0.126 (3)	−0.092 (3)
F10	0.110 (3)	0.092 (3)	0.193 (5)	0.008 (2)	−0.112 (3)	−0.036 (3)
F9	0.110 (3)	0.092 (3)	0.193 (5)	0.008 (2)	−0.112 (3)	−0.036 (3)

### Poly[[{µ-7,16-[3,3'-(diazene-1,2-diyl)bis(1,3-phenylenecarbonyl)]-1,4,10,13-\ tetraoxa-7,16-diazacyclooctadecane}bis{tetrakis[3,5-bis(trifluoromethyl)\ phenyl]borato}disodium] benzene disolvate] (2) Geometric parameters (Å, º)

**Table:**

Na1—O6	2.344 (2)	C43—C44	1.391 (4)
Na1—O5	2.313 (2)	C43—C48	1.406 (4)
Na1—O4	2.334 (2)	C43—B1	1.644 (4)
Na1—O3	2.366 (2)	C84—H84	0.9500
Na1—F45^i^	2.896 (3)	C84—C85	1.398 (4)
Na1—F6	2.362 (3)	C85—C86	1.384 (4)
Na2—F1	2.489 (2)	C85—C90	1.502 (4)
Na2—F2	2.723 (2)	C35—C40	1.398 (4)
Na2—F28^ii^	2.482 (2)	C35—B1	1.647 (4)
Na2—F27^ii^	2.729 (3)	F6—C27	1.314 (5)
Na2—F48^i^	2.584 (2)	C89—H89	0.9500
Na2—O1^iii^	2.275 (2)	C89—C87	1.393 (4)
Na2—F41^iv^	2.979 (2)	C6—H6	0.9500
Na2—O2	2.194 (2)	C6—C5	1.383 (4)
F1—C65	1.348 (4)	C6—C7	1.402 (4)
F49—C58	1.321 (3)	C86—H86	0.9500
F42—C66	1.338 (3)	C86—C87	1.389 (4)
F2—C65	1.348 (3)	C45—C44	1.395 (4)
F32—C73	1.335 (4)	C45—C46	1.380 (4)
F25—C90	1.335 (4)	C45—C50	1.490 (4)
F28—C74	1.337 (4)	C44—H44	0.9500
F31—C73	1.341 (3)	C78—H78	0.9500
F27—C74	1.341 (3)	C78—C77	1.379 (4)
F48—C58	1.354 (3)	C5—H5	0.9500
F3—C65	1.342 (3)	C26—H26A	0.9900
O1—C1	1.234 (3)	C26—H26B	0.9900
O6—C16	1.432 (3)	C26—C25	1.522 (4)
O6—C17	1.424 (4)	C62—H62	0.9500
F20—C50	1.333 (3)	C57—H57	0.9500
F50—C58	1.330 (3)	C57—C55	1.397 (4)
F30—C73	1.336 (4)	C77—C82	1.500 (4)
F26—C90	1.334 (4)	C47—C46	1.386 (4)
F23—C49	1.312 (4)	C47—C48	1.391 (4)
F18—C50	1.335 (4)	C47—C49	1.492 (4)
F17—C42	1.333 (4)	C30—C31	1.394 (4)
F35—C81	1.339 (3)	C30—C29	1.395 (4)
F19—C50	1.336 (3)	C30—B1	1.642 (4)
F44—C66	1.330 (3)	C46—H46	0.9500
F46—C56	1.317 (4)	C48—H48	0.9500
O5—C19	1.424 (3)	C10—C9	1.384 (4)
O5—C18	1.431 (3)	C10—C11	1.489 (4)
F33—C81	1.314 (3)	C10—C12	1.396 (4)
F41—C88	1.329 (5)	C19—H19A	0.9900
F40—C88	1.356 (4)	C19—H19B	0.9900
F34—C81	1.325 (3)	C7—H7	0.9500
F29—C74	1.320 (3)	C21—H21A	0.9900
F43—C66	1.327 (3)	C21—H21B	0.9900
O4—C22	1.442 (3)	C21—C22	1.509 (4)
O4—C23	1.447 (4)	C9—H9	0.9500
F24—C90	1.336 (3)	C37—C42	1.492 (4)
F15—C42	1.331 (4)	C37—C38	1.381 (5)
F47—C56	1.321 (4)	C16—H16A	0.9900
O3—C25	1.443 (4)	C16—H16B	0.9900
O3—C24	1.421 (4)	C16—C15	1.515 (4)
F37—C82	1.307 (4)	C18—H18A	0.9900
O2—C11	1.241 (3)	C18—H18B	0.9900
F16—C42	1.341 (4)	C18—C17	1.501 (4)
F21—C49	1.327 (4)	C88—C87	1.529 (5)
F39—C88	1.307 (4)	C22—H22A	0.9900
F45—C56	1.362 (4)	C22—H22B	0.9900
F36—C82	1.326 (5)	C25—H25A	0.9900
N3—C1	1.362 (3)	C25—H25B	0.9900
N3—C20	1.481 (3)	C31—H31	0.9500
N3—C21	1.467 (3)	C31—C32	1.393 (4)
N2—N1	1.255 (3)	C29—H29	0.9500
N2—C4	1.419 (3)	C29—C28	1.394 (4)
N1—C8	1.433 (4)	C12—H12	0.9500
F51—C34	1.339 (5)	C12—C13	1.385 (5)
N4—C26	1.471 (3)	C14—H14	0.9500
N4—C11	1.350 (4)	C14—C13	1.386 (5)
N4—C15	1.464 (4)	F7—C27	1.272 (6)
F22—C49	1.358 (4)	C82—F38	1.301 (4)
C79—C81	1.493 (4)	C55—C56	1.505 (4)
C79—C80	1.389 (3)	C15—H15A	0.9900
C79—C78	1.394 (4)	C15—H15B	0.9900
C61—C60	1.397 (3)	C32—C33	1.378 (5)
C61—C62	1.385 (4)	C32—C34	1.472 (5)
C61—C66	1.495 (4)	C17—H17A	0.9900
C68—H68	0.9500	C17—H17B	0.9900
C68—C67	1.399 (3)	F53—C34	1.218 (6)
C68—C69	1.390 (3)	C28—C33	1.369 (5)
C2—C1	1.492 (4)	C28—C27	1.493 (5)
C2—C3	1.386 (4)	C13—H13	0.9500
C2—C7	1.392 (4)	C23—H23A	0.9900
C53—C58	1.493 (3)	C23—H23B	0.9900
C53—C54	1.380 (4)	C23—C24	1.500 (5)
C53—C52	1.385 (4)	C40—H40	0.9500
C59—C60	1.394 (3)	C40—C39	1.395 (5)
C59—C64	1.403 (3)	C24—H24A	0.9900
C59—B2	1.644 (4)	C24—H24B	0.9900
C71—C72	1.392 (3)	C33—H33	0.9500
C71—C70	1.381 (4)	F8—C27	1.287 (6)
C71—C73	1.500 (4)	C38—H38	0.9500
C75—C76	1.392 (3)	C38—C39	1.383 (5)
C75—C80	1.406 (3)	C34—F4	1.451 (6)
C75—B2	1.640 (3)	C34—F5	1.290 (9)
C60—H60	0.9500	C34—F54	1.359 (8)
C74—C69	1.485 (4)	C34—F52	1.566 (7)
C83—C84	1.396 (3)	C39—C41	1.528 (6)
C83—C89	1.406 (4)	F12—C41	1.174 (16)
C83—B2	1.644 (4)	C95—H95	0.9500
C63—C64	1.388 (4)	C95—C94	1.3900
C63—C65	1.491 (4)	C95—C96	1.3900
C63—C62	1.384 (4)	C94—H94	0.9500
C72—H72	0.9500	C94—C93	1.3900
C72—C67	1.395 (3)	C93—H93	0.9500
C76—H76	0.9500	C93—C92	1.3900
C76—C77	1.394 (4)	C92—H92	0.9500
C67—B2	1.641 (3)	C92—C91	1.3900
C54—H54	0.9500	C91—H91	0.9500
C54—C55	1.387 (4)	C91—C96	1.3900
C4—C3	1.384 (4)	C96—H96	0.9500
C4—C5	1.389 (4)	C98—H98	0.9500
C64—H64	0.9500	C98—C99	1.3900
C80—H80	0.9500	C98—C97	1.3900
C70—H70	0.9500	C99—H99	0.9500
C70—C69	1.379 (4)	C99—C100	1.3900
C36—H36	0.9500	C100—H100	0.9500
C36—C35	1.393 (4)	C100—C101	1.3900
C36—C37	1.399 (4)	C101—H101	0.9500
C3—H3	0.9500	C101—C102	1.3900
C8—C9	1.390 (4)	C102—H102	0.9500
C8—C14	1.386 (4)	C102—C97	1.3900
C51—C52	1.410 (4)	C97—H97	0.9500
C51—C57	1.393 (4)	C41—F11	1.420 (10)
C51—B1	1.644 (4)	C41—F14	1.352 (6)
C20—H20A	0.9900	C41—F13	0.895 (16)
C20—H20B	0.9900	C41—F10	1.407 (15)
C20—C19	1.515 (3)	C41—F9	1.353 (9)
C52—H52	0.9500		
			
O6—Na1—O3	79.01 (7)	O5—C19—C20	114.7 (2)
O6—Na1—F45^i^	86.19 (8)	O5—C19—H19A	108.6
O6—Na1—F6	126.92 (14)	O5—C19—H19B	108.6
O5—Na1—O6	73.07 (7)	C20—C19—H19A	108.6
O5—Na1—O4	95.05 (8)	C20—C19—H19B	108.6
O5—Na1—O3	124.56 (9)	H19A—C19—H19B	107.6
O5—Na1—F45^i^	143.01 (9)	C2—C7—C6	119.5 (2)
O5—Na1—F6	93.62 (10)	C2—C7—H7	120.2
O4—Na1—O6	136.59 (9)	C6—C7—H7	120.2
O4—Na1—O3	74.14 (8)	N3—C21—H21A	109.1
O4—Na1—F45^i^	120.38 (9)	N3—C21—H21B	109.1
O4—Na1—F6	94.72 (14)	N3—C21—C22	112.7 (2)
O3—Na1—F45^i^	78.95 (8)	H21A—C21—H21B	107.8
F6—Na1—O3	140.51 (12)	C22—C21—H21A	109.1
F6—Na1—F45^i^	74.53 (10)	C22—C21—H21B	109.1
F1—Na2—F2	48.28 (6)	F23—C49—F21	108.6 (3)
F1—Na2—F27^ii^	137.74 (7)	F23—C49—F22	104.3 (3)
F1—Na2—F48^i^	128.91 (8)	F23—C49—C47	114.3 (2)
F1—Na2—F41^iv^	78.10 (7)	F21—C49—F22	104.4 (3)
F2—Na2—F27^ii^	133.04 (7)	F21—C49—C47	112.9 (2)
F2—Na2—F41^iv^	124.83 (7)	F22—C49—C47	111.5 (3)
F28^ii^—Na2—F1	156.07 (9)	F42—C66—C61	111.8 (2)
F28^ii^—Na2—F2	152.66 (8)	F44—C66—F42	105.9 (2)
F28^ii^—Na2—F27^ii^	47.46 (6)	F44—C66—C61	112.4 (2)
F28^ii^—Na2—F48^i^	73.25 (8)	F43—C66—F42	106.5 (2)
F28^ii^—Na2—F41^iv^	78.21 (7)	F43—C66—F44	107.2 (2)
F27^ii^—Na2—F41^iv^	93.40 (7)	F43—C66—C61	112.6 (2)
F48^i^—Na2—F2	80.67 (7)	C8—C9—H9	119.9
F48^i^—Na2—F27^ii^	75.86 (8)	C10—C9—C8	120.3 (2)
F48^i^—Na2—F41^iv^	149.14 (8)	C10—C9—H9	119.9
O1^iii^—Na2—F1	77.07 (7)	O2—C11—N4	121.5 (3)
O1^iii^—Na2—F2	72.55 (7)	O2—C11—C10	120.3 (3)
O1^iii^—Na2—F28^ii^	115.29 (8)	N4—C11—C10	117.7 (2)
O1^iii^—Na2—F27^ii^	67.89 (7)	C36—C37—C42	120.6 (3)
O1^iii^—Na2—F48^i^	91.04 (8)	C38—C37—C36	121.1 (3)
O1^iii^—Na2—F41^iv^	111.92 (8)	C38—C37—C42	118.3 (3)
O2—Na2—F1	92.83 (8)	O6—C16—H16A	109.6
O2—Na2—F2	87.51 (8)	O6—C16—H16B	109.6
O2—Na2—F28^ii^	80.80 (8)	O6—C16—C15	110.2 (2)
O2—Na2—F27^ii^	127.44 (8)	H16A—C16—H16B	108.1
O2—Na2—F48^i^	81.57 (8)	C15—C16—H16A	109.6
O2—Na2—O1^iii^	159.66 (9)	C15—C16—H16B	109.6
O2—Na2—F41^iv^	82.53 (9)	O5—C18—H18A	109.9
C65—F1—Na2	108.00 (16)	O5—C18—H18B	109.9
C65—F2—Na2	96.66 (17)	O5—C18—C17	108.8 (2)
C74—F28—Na2^v^	110.53 (16)	H18A—C18—H18B	108.3
C74—F27—Na2^v^	98.11 (17)	C17—C18—H18A	109.9
C58—F48—Na2^i^	148.09 (16)	C17—C18—H18B	109.9
C1—O1—Na2^vi^	148.68 (18)	F32—C73—F31	106.2 (2)
C16—O6—Na1	123.57 (19)	F32—C73—F30	106.5 (2)
C17—O6—Na1	110.34 (16)	F32—C73—C71	112.6 (2)
C17—O6—C16	112.0 (2)	F31—C73—C71	112.2 (2)
C19—O5—Na1	133.32 (16)	F30—C73—F31	106.6 (2)
C19—O5—C18	114.8 (2)	F30—C73—C71	112.3 (2)
C18—O5—Na1	111.32 (16)	F20—C50—F18	106.2 (3)
C88—F41—Na2^iv^	129.6 (2)	F20—C50—F19	106.7 (2)
C22—O4—Na1	137.77 (17)	F20—C50—C45	112.6 (2)
C22—O4—C23	110.9 (2)	F18—C50—F19	105.5 (2)
C23—O4—Na1	110.73 (17)	F18—C50—C45	112.3 (2)
C25—O3—Na1	127.28 (18)	F19—C50—C45	113.0 (2)
C24—O3—Na1	99.11 (17)	F41—C88—F40	105.4 (3)
C24—O3—C25	116.1 (2)	F41—C88—C87	113.0 (3)
C11—O2—Na2	174.3 (2)	F40—C88—C87	109.2 (3)
C56—F45—Na1^i^	155.4 (2)	F39—C88—F41	106.0 (4)
C1—N3—C20	119.3 (2)	F39—C88—F40	110.1 (3)
C1—N3—C21	117.1 (2)	F39—C88—C87	112.8 (3)
C21—N3—C20	119.5 (2)	C89—C87—C88	119.6 (3)
N1—N2—C4	117.2 (2)	C86—C87—C89	120.5 (3)
N2—N1—C8	109.7 (2)	C86—C87—C88	119.9 (3)
C11—N4—C26	123.8 (2)	O4—C22—C21	112.8 (2)
C11—N4—C15	118.1 (2)	O4—C22—H22A	109.0
C15—N4—C26	117.1 (2)	O4—C22—H22B	109.0
C80—C79—C81	121.3 (2)	C21—C22—H22A	109.0
C80—C79—C78	120.5 (2)	C21—C22—H22B	109.0
C78—C79—C81	118.2 (2)	H22A—C22—H22B	107.8
F35—C81—C79	112.8 (2)	O3—C25—C26	111.0 (2)
F33—C81—F35	104.6 (2)	O3—C25—H25A	109.4
F33—C81—F34	107.4 (3)	O3—C25—H25B	109.4
F33—C81—C79	113.0 (2)	C26—C25—H25A	109.4
F34—C81—F35	104.7 (2)	C26—C25—H25B	109.4
F34—C81—C79	113.6 (2)	H25A—C25—H25B	108.0
C60—C61—C66	119.8 (2)	C30—C31—H31	118.8
C62—C61—C60	120.4 (2)	C32—C31—C30	122.3 (3)
C62—C61—C66	119.8 (2)	C32—C31—H31	118.8
C67—C68—H68	118.9	C30—C29—H29	119.2
C69—C68—H68	118.9	C28—C29—C30	121.6 (3)
C69—C68—C67	122.1 (2)	C28—C29—H29	119.2
C3—C2—C1	117.2 (2)	F25—C90—F24	105.8 (3)
C3—C2—C7	119.2 (2)	F25—C90—C85	112.3 (2)
C7—C2—C1	123.3 (2)	F26—C90—F25	105.1 (3)
C54—C53—C58	118.7 (2)	F26—C90—F24	107.8 (3)
C54—C53—C52	121.5 (2)	F26—C90—C85	112.9 (2)
C52—C53—C58	119.7 (2)	F24—C90—C85	112.5 (3)
C60—C59—C64	115.6 (2)	C10—C12—H12	120.3
C60—C59—B2	125.0 (2)	C13—C12—C10	119.4 (3)
C64—C59—B2	119.3 (2)	C13—C12—H12	120.3
C72—C71—C73	119.5 (2)	C8—C14—H14	120.3
C70—C71—C72	121.0 (2)	C8—C14—C13	119.4 (3)
C70—C71—C73	119.5 (2)	C13—C14—H14	120.3
C76—C75—C80	115.2 (2)	F37—C82—F36	104.9 (3)
C76—C75—B2	125.6 (2)	F37—C82—C77	113.0 (3)
C80—C75—B2	119.0 (2)	F36—C82—C77	111.6 (3)
F49—C58—F48	105.2 (2)	F38—C82—F37	107.5 (3)
F49—C58—F50	108.2 (2)	F38—C82—F36	105.4 (4)
F49—C58—C53	114.1 (2)	F38—C82—C77	113.7 (3)
F48—C58—C53	111.7 (2)	C54—C55—C57	120.8 (3)
F50—C58—F48	103.5 (2)	C54—C55—C56	118.5 (3)
F50—C58—C53	113.3 (2)	C57—C55—C56	120.7 (3)
C61—C60—H60	118.6	N4—C15—C16	110.4 (2)
C59—C60—C61	122.8 (2)	N4—C15—H15A	109.6
C59—C60—H60	118.6	N4—C15—H15B	109.6
O1—C1—N3	122.1 (2)	C16—C15—H15A	109.6
O1—C1—C2	120.2 (2)	C16—C15—H15B	109.6
N3—C1—C2	117.1 (2)	H15A—C15—H15B	108.1
F28—C74—F27	103.9 (2)	C31—C32—C34	120.9 (3)
F28—C74—C69	112.8 (2)	C33—C32—C31	120.6 (3)
F27—C74—C69	112.9 (2)	C33—C32—C34	118.4 (3)
F29—C74—F28	107.2 (3)	O6—C17—C18	109.0 (2)
F29—C74—F27	106.2 (2)	O6—C17—H17A	109.9
F29—C74—C69	113.2 (2)	O6—C17—H17B	109.9
C84—C83—C89	115.3 (2)	C18—C17—H17A	109.9
C84—C83—B2	124.1 (2)	C18—C17—H17B	109.9
C89—C83—B2	120.6 (2)	H17A—C17—H17B	108.3
C64—C63—C65	118.6 (2)	C29—C28—C27	119.2 (3)
C62—C63—C64	121.6 (2)	C33—C28—C29	121.5 (3)
C62—C63—C65	119.7 (2)	C33—C28—C27	119.3 (3)
C71—C72—H72	118.9	C12—C13—C14	121.0 (3)
C71—C72—C67	122.2 (2)	C12—C13—H13	119.5
C67—C72—H72	118.9	C14—C13—H13	119.5
C75—C76—H76	118.9	F46—C56—F47	107.1 (3)
C75—C76—C77	122.3 (2)	F46—C56—F45	105.8 (3)
C77—C76—H76	118.9	F46—C56—C55	113.1 (3)
C68—C67—B2	118.9 (2)	F47—C56—F45	105.9 (3)
C72—C67—C68	115.6 (2)	F47—C56—C55	112.5 (3)
C72—C67—B2	125.5 (2)	F45—C56—C55	111.8 (3)
C53—C54—H54	121.2	O4—C23—H23A	109.4
C53—C54—C55	117.6 (2)	O4—C23—H23B	109.4
C55—C54—H54	121.2	O4—C23—C24	111.1 (3)
C3—C4—N2	111.8 (2)	H23A—C23—H23B	108.0
C3—C4—C5	120.8 (2)	C24—C23—H23A	109.4
C5—C4—N2	127.4 (2)	C24—C23—H23B	109.4
C59—C64—H64	119.1	C35—C40—H40	118.7
C63—C64—C59	121.7 (2)	C39—C40—C35	122.5 (3)
C63—C64—H64	119.1	C39—C40—H40	118.7
C79—C80—C75	122.7 (2)	O3—C24—C23	112.3 (3)
C79—C80—H80	118.6	O3—C24—H24A	109.1
C75—C80—H80	118.6	O3—C24—H24B	109.1
C71—C70—H70	121.1	C23—C24—H24A	109.1
C69—C70—C71	117.9 (2)	C23—C24—H24B	109.1
C69—C70—H70	121.1	H24A—C24—H24B	107.9
C35—C36—H36	118.9	C32—C33—H33	120.9
C35—C36—C37	122.2 (3)	C28—C33—C32	118.1 (3)
C37—C36—H36	118.9	C28—C33—H33	120.9
C2—C3—H3	119.6	C59—B2—C83	110.41 (19)
C4—C3—C2	120.8 (2)	C75—B2—C59	108.09 (19)
C4—C3—H3	119.6	C75—B2—C83	112.81 (19)
C9—C8—N1	121.7 (2)	C75—B2—C67	109.72 (19)
C14—C8—N1	118.1 (3)	C67—B2—C59	109.55 (19)
C14—C8—C9	120.1 (3)	C67—B2—C83	106.24 (19)
C52—C51—B1	119.8 (2)	F17—C42—F16	105.5 (3)
C57—C51—C52	115.2 (2)	F17—C42—C37	112.9 (3)
C57—C51—B1	125.0 (2)	F15—C42—F17	106.5 (3)
N3—C20—H20A	108.2	F15—C42—F16	106.4 (3)
N3—C20—H20B	108.2	F15—C42—C37	113.1 (3)
N3—C20—C19	116.4 (2)	F16—C42—C37	111.9 (3)
H20A—C20—H20B	107.4	C37—C38—H38	121.1
C19—C20—H20A	108.2	C37—C38—C39	117.8 (3)
C19—C20—H20B	108.2	C39—C38—H38	121.1
C68—C69—C74	117.7 (2)	F51—C34—C32	110.7 (3)
C70—C69—C68	121.1 (2)	F51—C34—F4	95.2 (4)
C70—C69—C74	121.2 (2)	C32—C34—F52	106.7 (4)
C53—C52—C51	122.2 (2)	F53—C34—F51	114.6 (5)
C53—C52—H52	118.9	F53—C34—C32	119.7 (4)
C51—C52—H52	118.9	F53—C34—F4	106.0 (5)
C44—C43—C48	115.9 (2)	F4—C34—C32	107.3 (4)
C44—C43—B1	124.4 (2)	F5—C34—C32	129.4 (6)
C48—C43—B1	119.7 (2)	F5—C34—F54	113.0 (6)
C83—C84—H84	118.8	F5—C34—F52	96.6 (6)
C83—C84—C85	122.5 (2)	F54—C34—C32	113.5 (5)
C85—C84—H84	118.8	F54—C34—F52	84.3 (5)
C84—C85—C90	119.2 (3)	F6—C27—C28	112.6 (3)
C86—C85—C84	120.8 (2)	F7—C27—F6	104.7 (5)
C86—C85—C90	120.0 (2)	F7—C27—C28	115.7 (3)
C36—C35—C40	115.5 (3)	F7—C27—F8	104.2 (4)
C36—C35—B1	124.5 (2)	F8—C27—F6	104.2 (4)
C40—C35—B1	120.0 (3)	F8—C27—C28	114.3 (4)
C27—F6—Na1	146.5 (3)	C40—C39—C41	118.9 (4)
C83—C89—H89	118.7	C38—C39—C40	120.7 (3)
C87—C89—C83	122.7 (2)	C38—C39—C41	120.3 (3)
C87—C89—H89	118.7	C51—B1—C35	107.8 (2)
C5—C6—H6	119.5	C43—B1—C51	110.6 (2)
C5—C6—C7	121.1 (3)	C43—B1—C35	109.9 (2)
C7—C6—H6	119.5	C30—B1—C51	108.2 (2)
C85—C86—H86	120.9	C30—B1—C43	108.1 (2)
C85—C86—C87	118.2 (2)	C30—B1—C35	112.2 (2)
C87—C86—H86	120.9	C94—C95—H95	120.0
F1—C65—F2	105.2 (2)	C94—C95—C96	120.0
F1—C65—C63	113.3 (2)	C96—C95—H95	120.0
F2—C65—C63	112.9 (3)	C95—C94—H94	120.0
F3—C65—F1	105.0 (2)	C95—C94—C93	120.0
F3—C65—F2	107.5 (2)	C93—C94—H94	120.0
F3—C65—C63	112.3 (2)	C94—C93—H93	120.0
C44—C45—C50	118.7 (2)	C92—C93—C94	120.0
C46—C45—C44	120.9 (2)	C92—C93—H93	120.0
C46—C45—C50	120.3 (2)	C93—C92—H92	120.0
C43—C44—C45	122.2 (2)	C91—C92—C93	120.0
C43—C44—H44	118.9	C91—C92—H92	120.0
C45—C44—H44	118.9	C92—C91—H91	120.0
C79—C78—H78	121.2	C92—C91—C96	120.0
C77—C78—C79	117.6 (2)	C96—C91—H91	120.0
C77—C78—H78	121.2	C95—C96—H96	120.0
C4—C5—H5	120.7	C91—C96—C95	120.0
C6—C5—C4	118.7 (3)	C91—C96—H96	120.0
C6—C5—H5	120.7	C99—C98—H98	120.0
N4—C26—H26A	108.3	C99—C98—C97	120.0
N4—C26—H26B	108.3	C97—C98—H98	120.0
N4—C26—C25	115.8 (2)	C98—C99—H99	120.0
H26A—C26—H26B	107.4	C100—C99—C98	120.0
C25—C26—H26A	108.3	C100—C99—H99	120.0
C25—C26—H26B	108.3	C99—C100—H100	120.0
C61—C62—H62	121.1	C99—C100—C101	120.0
C63—C62—C61	117.8 (2)	C101—C100—H100	120.0
C63—C62—H62	121.1	C100—C101—H101	120.0
C51—C57—H57	118.7	C102—C101—C100	120.0
C51—C57—C55	122.6 (2)	C102—C101—H101	120.0
C55—C57—H57	118.7	C101—C102—H102	120.0
C76—C77—C82	119.1 (3)	C101—C102—C97	120.0
C78—C77—C76	121.4 (2)	C97—C102—H102	120.0
C78—C77—C82	119.4 (3)	C98—C97—H97	120.0
C46—C47—C48	120.9 (2)	C102—C97—C98	120.0
C46—C47—C49	118.1 (2)	C102—C97—H97	120.0
C48—C47—C49	121.0 (2)	F12—C41—C39	117.2 (14)
C31—C30—C29	115.8 (3)	F12—C41—F10	65.8 (11)
C31—C30—B1	120.2 (2)	F11—C41—C39	105.2 (5)
C29—C30—B1	123.9 (3)	F14—C41—C39	111.8 (4)
C45—C46—C47	118.1 (2)	F14—C41—F11	109.4 (7)
C45—C46—H46	120.9	F14—C41—F9	106.1 (6)
C47—C46—H46	120.9	F13—C41—C39	129.8 (12)
C43—C48—H48	119.1	F13—C41—F12	85.3 (14)
C47—C48—C43	121.9 (2)	F13—C41—F10	113.4 (13)
C47—C48—H48	119.1	F10—C41—C39	116.8 (7)
C9—C10—C11	116.3 (2)	F9—C41—C39	111.3 (5)
C9—C10—C12	119.8 (3)	F9—C41—F11	113.1 (5)
C12—C10—C11	123.7 (3)		
			
Na1—O6—C16—C15	71.4 (3)	C89—C83—B2—C75	−46.6 (3)
Na1—O6—C17—C18	−44.2 (3)	C89—C83—B2—C67	73.7 (3)
Na1—O5—C19—C20	−89.8 (3)	C86—C85—C90—F25	−108.0 (3)
Na1—O5—C18—C17	−43.9 (3)	C86—C85—C90—F26	133.4 (3)
Na1—O4—C22—C21	34.6 (4)	C86—C85—C90—F24	11.2 (4)
Na1—O4—C23—C24	12.6 (3)	C65—C63—C64—C59	174.5 (2)
Na1—O3—C25—C26	112.5 (2)	C65—C63—C62—C61	−176.5 (2)
Na1—O3—C24—C23	62.1 (3)	C44—C43—C48—C47	−0.3 (4)
Na1^i^—F45—C56—F46	−108.8 (4)	C44—C43—B1—C51	−15.1 (3)
Na1^i^—F45—C56—F47	4.7 (6)	C44—C43—B1—C35	103.8 (3)
Na1^i^—F45—C56—C55	127.6 (4)	C44—C43—B1—C30	−133.4 (3)
Na1—F6—C27—F7	71.2 (8)	C44—C45—C46—C47	0.0 (4)
Na1—F6—C27—C28	−162.3 (5)	C44—C45—C50—F20	53.8 (3)
Na1—F6—C27—F8	−37.9 (9)	C44—C45—C50—F18	−66.0 (3)
Na2—F1—C65—F2	−13.6 (2)	C44—C45—C50—F19	174.8 (2)
Na2—F1—C65—F3	−126.82 (17)	C78—C79—C81—F35	−65.9 (3)
Na2—F1—C65—C63	110.2 (2)	C78—C79—C81—F33	52.5 (3)
Na2—F2—C65—F1	11.8 (2)	C78—C79—C81—F34	175.1 (3)
Na2—F2—C65—F3	123.3 (2)	C78—C79—C80—C75	−1.7 (4)
Na2—F2—C65—C63	−112.2 (2)	C78—C77—C82—F37	131.1 (3)
Na2^v^—F28—C74—F27	−0.9 (3)	C78—C77—C82—F36	−110.9 (3)
Na2^v^—F28—C74—F29	111.25 (19)	C78—C77—C82—F38	8.1 (5)
Na2^v^—F28—C74—C69	−123.6 (2)	C5—C4—C3—C2	−0.8 (4)
Na2^v^—F27—C74—F28	0.8 (2)	C5—C6—C7—C2	−0.1 (4)
Na2^v^—F27—C74—F29	−112.1 (2)	C26—N4—C11—O2	−157.6 (3)
Na2^v^—F27—C74—C69	123.3 (2)	C26—N4—C11—C10	30.0 (4)
Na2^i^—F48—C58—F49	−31.3 (4)	C26—N4—C15—C16	−124.3 (3)
Na2^i^—F48—C58—F50	82.1 (4)	C62—C61—C60—C59	0.9 (4)
Na2^i^—F48—C58—C53	−155.6 (3)	C62—C61—C66—F42	102.7 (3)
Na2^vi^—O1—C1—N3	−166.1 (2)	C62—C61—C66—F44	−138.4 (3)
Na2^vi^—O1—C1—C2	5.3 (5)	C62—C61—C66—F43	−17.1 (4)
Na2^iv^—F41—C88—F40	−86.5 (3)	C62—C63—C64—C59	−1.0 (4)
Na2^iv^—F41—C88—F39	30.3 (3)	C62—C63—C65—F1	−138.5 (3)
Na2^iv^—F41—C88—C87	154.33 (19)	C62—C63—C65—F2	−19.0 (4)
F28—C74—C69—C68	−48.5 (3)	C62—C63—C65—F3	102.7 (3)
F28—C74—C69—C70	132.6 (3)	C57—C51—C52—C53	−1.1 (4)
F27—C74—C69—C68	−165.9 (2)	C57—C51—B1—C43	−107.6 (3)
F27—C74—C69—C70	15.2 (4)	C57—C51—B1—C35	132.3 (3)
O6—C16—C15—N4	57.6 (3)	C57—C51—B1—C30	10.7 (4)
O5—C18—C17—O6	59.6 (3)	C57—C55—C56—F46	−27.1 (4)
F41—C88—C87—C89	48.2 (4)	C57—C55—C56—F47	−148.6 (3)
F41—C88—C87—C86	−129.1 (3)	C57—C55—C56—F45	92.3 (4)
F40—C88—C87—C89	−68.7 (4)	C30—C31—C32—C33	−0.8 (5)
F40—C88—C87—C86	114.0 (3)	C30—C31—C32—C34	175.8 (3)
F29—C74—C69—C68	73.4 (3)	C30—C29—C28—C33	−1.2 (5)
F29—C74—C69—C70	−105.5 (3)	C30—C29—C28—C27	178.5 (3)
O4—C23—C24—O3	−54.2 (4)	C46—C45—C44—C43	−0.5 (4)
F39—C88—C87—C89	168.5 (3)	C46—C45—C50—F20	−127.6 (3)
F39—C88—C87—C86	−8.8 (5)	C46—C45—C50—F18	112.6 (3)
N3—C20—C19—O5	62.3 (3)	C46—C45—C50—F19	−6.6 (4)
N3—C21—C22—O4	61.4 (3)	C46—C47—C48—C43	−0.1 (4)
N2—N1—C8—C9	−15.6 (3)	C46—C47—C49—F23	−174.8 (3)
N2—N1—C8—C14	161.0 (2)	C46—C47—C49—F21	−49.9 (4)
N2—C4—C3—C2	−179.5 (2)	C46—C47—C49—F22	67.2 (3)
N2—C4—C5—C6	178.5 (3)	C48—C43—C44—C45	0.6 (4)
N1—N2—C4—C3	−176.1 (2)	C48—C43—B1—C51	166.1 (2)
N1—N2—C4—C5	5.2 (4)	C48—C43—B1—C35	−75.0 (3)
N1—C8—C9—C10	174.0 (2)	C48—C43—B1—C30	47.8 (3)
N1—C8—C14—C13	−175.2 (3)	C48—C47—C46—C45	0.3 (4)
N4—C26—C25—O3	−67.3 (3)	C48—C47—C49—F23	6.5 (4)
C79—C78—C77—C76	2.0 (4)	C48—C47—C49—F21	131.3 (3)
C79—C78—C77—C82	179.5 (3)	C48—C47—C49—F22	−111.6 (3)
C81—C79—C80—C75	176.4 (2)	C10—C12—C13—C14	−0.3 (5)
C81—C79—C78—C77	−179.7 (2)	C19—O5—C18—C17	143.3 (2)
C68—C67—B2—C59	−51.6 (3)	C7—C2—C1—O1	49.4 (4)
C68—C67—B2—C75	−170.1 (2)	C7—C2—C1—N3	−138.7 (3)
C68—C67—B2—C83	67.7 (3)	C7—C2—C3—C4	1.2 (4)
C53—C54—C55—C57	−3.7 (4)	C7—C6—C5—C4	0.5 (4)
C53—C54—C55—C56	173.3 (3)	C21—N3—C1—O1	10.0 (4)
C71—C72—C67—C68	2.7 (4)	C21—N3—C1—C2	−161.7 (2)
C71—C72—C67—B2	−176.3 (2)	C21—N3—C20—C19	43.0 (3)
C71—C70—C69—C68	0.5 (4)	C49—C47—C46—C45	−178.5 (3)
C71—C70—C69—C74	179.4 (2)	C49—C47—C48—C43	178.6 (3)
C75—C76—C77—C78	0.9 (4)	C66—C61—C60—C59	−179.0 (2)
C75—C76—C77—C82	−176.6 (3)	C66—C61—C62—C63	−179.0 (2)
C58—C53—C54—C55	−173.0 (3)	C9—C8—C14—C13	1.5 (4)
C58—C53—C52—C51	175.3 (2)	C9—C10—C11—O2	−121.0 (3)
C60—C61—C62—C63	1.0 (4)	C9—C10—C11—N4	51.4 (3)
C60—C61—C66—F42	−77.3 (3)	C9—C10—C12—C13	−0.8 (4)
C60—C61—C66—F44	41.6 (3)	C11—N4—C26—C25	−120.2 (3)
C60—C61—C66—F43	162.8 (2)	C11—N4—C15—C16	66.7 (3)
C60—C59—C64—C63	2.8 (3)	C11—C10—C9—C8	177.7 (2)
C60—C59—B2—C75	110.0 (2)	C11—C10—C12—C13	−176.0 (3)
C60—C59—B2—C83	−126.2 (2)	C37—C36—C35—C40	−0.8 (4)
C60—C59—B2—C67	−9.5 (3)	C37—C36—C35—B1	179.0 (3)
C1—N3—C20—C19	−160.5 (2)	C37—C38—C39—C40	1.7 (7)
C1—N3—C21—C22	65.0 (3)	C37—C38—C39—C41	−179.9 (5)
C1—C2—C3—C4	174.8 (2)	C16—O6—C17—C18	174.0 (2)
C1—C2—C7—C6	−174.0 (2)	C18—O5—C19—C20	81.0 (3)
C83—C84—C85—C86	0.9 (4)	C73—C71—C72—C67	−179.4 (2)
C83—C84—C85—C90	−179.7 (2)	C73—C71—C70—C69	177.7 (2)
C83—C89—C87—C86	3.4 (4)	C50—C45—C44—C43	178.1 (2)
C83—C89—C87—C88	−173.9 (3)	C50—C45—C46—C47	−178.5 (2)
C72—C71—C70—C69	−1.6 (4)	C22—O4—C23—C24	−159.9 (3)
C72—C71—C73—F32	−30.8 (4)	C25—O3—C24—C23	−77.5 (3)
C72—C71—C73—F31	−150.5 (2)	C31—C30—C29—C28	1.8 (4)
C72—C71—C73—F30	89.4 (3)	C31—C30—B1—C51	−79.6 (3)
C72—C67—B2—C59	127.4 (2)	C31—C30—B1—C43	40.2 (3)
C72—C67—B2—C75	8.9 (3)	C31—C30—B1—C35	161.5 (2)
C72—C67—B2—C83	−113.3 (3)	C31—C32—C33—C28	1.5 (5)
C76—C75—C80—C79	4.4 (3)	C31—C32—C34—F51	131.9 (4)
C76—C75—B2—C59	116.1 (3)	C31—C32—C34—F53	−4.8 (7)
C76—C75—B2—C83	−6.2 (3)	C31—C32—C34—F4	−125.4 (4)
C76—C75—B2—C67	−124.5 (2)	C31—C32—C34—F5	150.4 (7)
C76—C77—C82—F37	−51.3 (4)	C31—C32—C34—F54	−54.3 (7)
C76—C77—C82—F36	66.7 (4)	C31—C32—C34—F52	36.7 (5)
C76—C77—C82—F38	−174.3 (4)	C29—C30—C31—C32	−0.8 (4)
C67—C68—C69—C74	−176.7 (2)	C29—C30—B1—C51	97.2 (3)
C67—C68—C69—C70	2.2 (4)	C29—C30—B1—C43	−143.0 (3)
C54—C53—C58—F49	−33.0 (3)	C29—C30—B1—C35	−21.7 (4)
C54—C53—C58—F48	86.1 (3)	C29—C28—C33—C32	−0.6 (5)
C54—C53—C58—F50	−157.4 (3)	C29—C28—C27—F6	−97.9 (5)
C54—C53—C52—C51	−1.4 (4)	C29—C28—C27—F7	22.4 (6)
C54—C55—C56—F46	155.9 (3)	C29—C28—C27—F8	143.4 (4)
C54—C55—C56—F47	34.4 (4)	C90—C85—C86—C87	179.7 (3)
C54—C55—C56—F45	−84.7 (4)	C12—C10—C9—C8	2.2 (4)
C4—N2—N1—C8	−177.7 (2)	C12—C10—C11—O2	54.3 (4)
C64—C59—C60—C61	−2.8 (3)	C12—C10—C11—N4	−133.2 (3)
C64—C59—B2—C75	−72.3 (3)	C14—C8—C9—C10	−2.6 (4)
C64—C59—B2—C83	51.6 (3)	C15—N4—C26—C25	71.5 (3)
C64—C59—B2—C67	168.2 (2)	C15—N4—C11—O2	10.6 (4)
C64—C63—C65—F1	45.8 (3)	C15—N4—C11—C10	−161.7 (2)
C64—C63—C65—F2	165.3 (2)	C17—O6—C16—C15	−152.6 (3)
C64—C63—C65—F3	−73.0 (3)	C23—O4—C22—C21	−155.9 (3)
C64—C63—C62—C61	−1.0 (4)	C40—C35—B1—C51	−50.9 (4)
C80—C79—C81—F35	116.0 (3)	C40—C35—B1—C43	−171.5 (3)
C80—C79—C81—F33	−125.7 (3)	C40—C35—B1—C30	68.2 (4)
C80—C79—C81—F34	−3.0 (4)	C40—C39—C41—F12	−83.4 (13)
C80—C79—C78—C77	−1.6 (4)	C40—C39—C41—F11	−64.0 (7)
C80—C75—C76—C77	−4.0 (4)	C40—C39—C41—F14	177.3 (6)
C80—C75—B2—C59	−68.2 (3)	C40—C39—C41—F13	169 (2)
C80—C75—B2—C83	169.5 (2)	C40—C39—C41—F10	−8.2 (14)
C80—C75—B2—C67	51.2 (3)	C40—C39—C41—F9	58.8 (7)
C70—C71—C72—C67	−0.1 (4)	C24—O3—C25—C26	−121.0 (3)
C70—C71—C73—F32	149.9 (2)	C33—C32—C34—F51	−51.4 (5)
C70—C71—C73—F31	30.1 (4)	C33—C32—C34—F53	171.9 (5)
C70—C71—C73—F30	−89.9 (3)	C33—C32—C34—F4	51.3 (5)
C36—C35—C40—C39	3.7 (6)	C33—C32—C34—F5	−32.9 (9)
C36—C35—B1—C51	129.4 (3)	C33—C32—C34—F54	122.4 (6)
C36—C35—B1—C43	8.8 (4)	C33—C32—C34—F52	−146.6 (4)
C36—C35—B1—C30	−111.5 (3)	C33—C28—C27—F6	81.7 (5)
C36—C37—C42—F17	37.6 (4)	C33—C28—C27—F7	−158.0 (5)
C36—C37—C42—F15	158.7 (3)	C33—C28—C27—F8	−37.0 (6)
C36—C37—C42—F16	−81.2 (4)	B2—C59—C60—C61	175.0 (2)
C36—C37—C38—C39	1.1 (6)	B2—C59—C64—C63	−175.1 (2)
C3—C2—C1—O1	−123.9 (3)	B2—C75—C76—C77	171.8 (2)
C3—C2—C1—N3	48.0 (3)	B2—C75—C80—C79	−171.7 (2)
C3—C2—C7—C6	−0.8 (4)	B2—C83—C84—C85	178.6 (2)
C3—C4—C5—C6	−0.1 (4)	B2—C83—C89—C87	179.2 (2)
C8—C14—C13—C12	−0.1 (5)	C42—C37—C38—C39	−176.2 (4)
C51—C57—C55—C54	1.3 (5)	C38—C37—C42—F17	−145.0 (3)
C51—C57—C55—C56	−175.6 (3)	C38—C37—C42—F15	−23.9 (4)
C20—N3—C1—O1	−147.1 (2)	C38—C37—C42—F16	96.2 (4)
C20—N3—C1—C2	41.2 (3)	C38—C39—C41—F12	98.1 (13)
C20—N3—C21—C22	−138.0 (2)	C38—C39—C41—F11	117.5 (5)
C69—C68—C67—C72	−3.8 (4)	C38—C39—C41—F14	−1.2 (10)
C69—C68—C67—B2	175.4 (2)	C38—C39—C41—F13	−10 (2)
C52—C53—C58—F49	150.2 (2)	C38—C39—C41—F10	173.3 (11)
C52—C53—C58—F48	−90.6 (3)	C38—C39—C41—F9	−119.6 (5)
C52—C53—C58—F50	25.8 (3)	C34—C32—C33—C28	−175.2 (3)
C52—C53—C54—C55	3.7 (4)	C27—C28—C33—C32	179.8 (3)
C52—C51—C57—C55	1.1 (4)	B1—C51—C52—C53	179.9 (2)
C52—C51—B1—C43	71.4 (3)	B1—C51—C57—C55	−179.9 (3)
C52—C51—B1—C35	−48.8 (3)	B1—C43—C44—C45	−178.2 (2)
C52—C51—B1—C30	−170.4 (2)	B1—C43—C48—C47	178.6 (2)
C84—C83—C89—C87	−3.3 (4)	B1—C35—C40—C39	−176.1 (4)
C84—C83—B2—C59	15.1 (3)	B1—C30—C31—C32	176.2 (3)
C84—C83—B2—C75	136.2 (2)	B1—C30—C29—C28	−175.1 (3)
C84—C83—B2—C67	−103.6 (3)	C95—C94—C93—C92	0.0
C84—C85—C86—C87	−0.9 (4)	C94—C95—C96—C91	0.0
C84—C85—C90—F25	72.6 (3)	C94—C93—C92—C91	0.0
C84—C85—C90—F26	−45.9 (4)	C93—C92—C91—C96	0.0
C84—C85—C90—F24	−168.1 (3)	C92—C91—C96—C95	0.0
C85—C86—C87—C89	−1.1 (4)	C96—C95—C94—C93	0.0
C85—C86—C87—C88	176.2 (3)	C98—C99—C100—C101	0.0
C35—C36—C37—C42	175.7 (3)	C99—C98—C97—C102	0.0
C35—C36—C37—C38	−1.6 (5)	C99—C100—C101—C102	0.0
C35—C40—C39—C38	−4.2 (7)	C100—C101—C102—C97	0.0
C35—C40—C39—C41	177.3 (5)	C101—C102—C97—C98	0.0
C89—C83—C84—C85	1.2 (4)	C97—C98—C99—C100	0.0
C89—C83—B2—C59	−167.6 (2)		

Symmetry codes: (i) −*x*+2, −*y*+1, −*z*+1; (ii) *x*+1, *y*, *z*; (iii) *x*, *y*−1, *z*; (iv) −*x*+1, −*y*, −*z*+2; (v) *x*−1, *y*, *z*; (vi) *x*, *y*+1, *z*.
